# Depletion of yeast PDK1 orthologs triggers a stress-like transcriptional response

**DOI:** 10.1186/s12864-015-1903-8

**Published:** 2015-09-21

**Authors:** Daniel Pastor-Flores, Jofre Ferrer-Dalmau, Anna Bahí, Martí Boleda, Ricardo M. Biondi, Antonio Casamayor

**Affiliations:** Research Group PhosphoSites, Medizinische Klinik I, Universitätsklinikum Frankfurt, Theodor-Stern-Kai 7, 60590 Frankfurt, Germany; Departament de Bioquímica i Biologia Molecular, Facultat de Veterinària, Universitat Autònoma de Barcelona, Cerdanyola 08193, Barcelona, Spain; Institut de Biotecnologia i Biomedicina, Universitat Autònoma de Barcelona, Cerdanyola 08193, Barcelona, Spain; Present address: Division of Redox Regulation, German Cancer Research Center, DKFZ-ZMBH Alliance, Im Neuenheimer Feld 280, 69120 Heidelberg, Germany; Laboratoire d’Ecologie Alpine (LECA), UMR 5553, CNRS-Université Joseph Fourie, BP 53, 38041 Grenoble, France

**Keywords:** Yeast, Pkh protein kinases, DNA Microarray, doxycycline-repressive promoter, Heat shock

## Abstract

**Background:**

Pkh proteins are the PDK1 orthologs in *S. cerevisiae*. They have redundant and essential activity and are responsible for the phosphorylation of several members of the AGC family of protein kinases. Pkh proteins have been involved in several cellular functions, including cell wall integrity and endocytosis. However the global expression changes caused by their depletion are still unknown.

**Results:**

A doxycycline-repressible tetO_7_ promoter driving the expression of *PKH2* in cells carrying deletions of the *PKH1* and *PKH3* genes allowed us to progressively deplete cells from Pkh proteins when treated with doxycycline. Global gene expression analysis indicate that depletion of Pkh results in the up-regulation of genes involved in the accumulation of glycogen and also of those related to stress responses. Moreover, genes involved in the ion transport were quickly down-regulated when the levels of Pkh decreased. The reduction in the mRNA levels required for protein translation, however, was only observed after longer doxycycline treatment (24 h). We uncovered that Pkh is important for the proper transcriptional response to heat shock, and is mostly required for the effects driven by the transcription factors Hsf1 and Msn2/Msn4, but is not required for down-regulation of the mRNA coding for ribosomal proteins.

**Conclusions:**

By using the tetO_7_ promoter we elucidated for the first time the transcriptomic changes directly or indirectly caused by progressive depletion of Pkh. Furthermore, this system enabled the characterization of the transcriptional response triggered by heat shock in wild-type and Pkh-depleted cells, showing that about 40 % of the observed expression changes were, to some degree, dependent on Pkh.

**Electronic supplementary material:**

The online version of this article (doi:10.1186/s12864-015-1903-8) contains supplementary material, which is available to authorized users.

## Background

The 3-phosphoinositide-dependent kinase (PDK1), a master kinase ubiquitously present in eukaryotic life forms, has critical and diverse roles in cells and organisms. In multicellular organisms, PDK1 phosphorylates its substrate PKB (also termed Akt) in response to increased levels of phosphatidylinositol (3,4,5) triphosphate, which triggers co-localization of the kinases mediated by their pleckstrin homology (PH) domains. Deregulation of PKB in mammals leads to important disorders such as cancer, diabetes, cardiovascular and neurological diseases. PDK1 also phosphorylates the activation loop of at least 20 other members of the so-called AGC family of protein kinases, including SGK, p70 S6 kinase, and PKC. However, this activity does not require the direct interaction of PDK1 with phosphoinositides [1–3]. This set of AGC kinases plays important cellular roles in all studied organisms. In mammals they are involved in cell growth, proliferation, differentiation and survival, tumor growth, aldosterone and insulin release, glucose metabolism, gastric acid secretion, regulation of ion transporters and channels, blood pressure, ribosome biogenesis, protein synthesis, cell cycle progression and metabolism, among other important functions [4, 5]. It has been demonstrated that in plants PDK1 is required for the activation of not less of 16 AGC kinases and, similar to what happens in animals, PDK1 regulates signaling pathways necessary for proper growth in normal and stress conditions [6, 7].

The yeast orthologs Pkh1 and Pkh2 proteins have a kinase domain that is 47.7 % identical to that of human PDK1 (73.5 % and 74.6 % of similarity, respectively). Pkh1 and Pkh2 have redundant and essential functions since cells carrying the double *pkh1* and *pkh2* deletions are not viable but any single mutant is viable [8]. Pkh3 is a third and more distantly related protein whose catalytic domain displays 42.9 % identity with that of PDK1 (65.6 % similar). The single *pkh3* deletion does not display an obvious phenotype [9]. Accordingly, and in contrast to what occurs with the *pkh1 pkh2* double-mutant cells, the *pkh1 pkh3* and *pkh2 pkh3* double-mutant strains grow normally. Lack of *PKH3*, however, exacerbates the growth defect of the *pkh1*^*D398G*^*pkh2* mutant at the restrictive temperature [10].

As in other organisms, yeast Pkh proteins exert pleiotropic effects by phosphorylating the activation loop of diverse AGC protein kinases and by the direct phosphorylation of other regulatory proteins. Among the identified substrates of the yeast Pkh kinases are the protein kinases Ypk1, Pkc1, Sch9 [11] and Tpk1, one of the catalytic subunits of PKA [10, 12]. Sch9 phosphorylation by Pkh regulates lifespan and oxidative stress sensitivity in a process that depends on sphingolipids [13, 14]. Phosphorylation of Pkc1 activates the Slt2 MAPK cascade and is crucial for maintaining cell wall integrity (CWI) [9, 15].

One of the more commonly used methods to identify the functions of a protein is to analyze the phenotypes caused by the lack of this protein. When redundant proteins are present it will be necessary to simultaneously remove all those redundant proteins, usually by deleting the corresponding genes. However, alternative strategies should be used when the elimination of the genes coding for the redundant proteins leads to non-viable cells. In the case of the essential family of Pkh protein kinases most of the information obtained has been acquired using a Pkh1^D398G^ temperature-sensitive allele in combination with deletion of the *PKH2* gene and incubation at the restrictive temperature of 37 °C. We have recently reported an alternative genetic strategy to deplete cells of Pkh activity circumventing the incubation at stressful temperatures [15]. In the newly generated strains the expression of *PKH2*, controlled by the tetO_7_ promoter, is decreased by addition of doxycycline. Therefore, elimination of the essential Pkh activity in this system can be achieved by deleting *PKH1* and by incubation of cells in the presence of doxycycline. We decided, however, to delete also the *PKH3* gene, since it was identified as a multicopy suppressor of the lethality caused when *pkh1*^*D398G*^*pkh2* cells were incubated at the restrictive temperature [9]. Our strategy allows the phenotypic analysis caused by depletion of Pkh with no need of incubation of cells at 37 °C, a temperature that triggers the activation of the CWI pathway. This approach has been successfully used to demonstrate the importance of Pkh in the activation of the CWI pathway in the absence of a heat shock [15].

The identification of the global changes in the expression profile caused by the lack of the studied protein is a more comprehensive approach to identify potential new functional roles for that protein. As expected for the case of redundant proteins, the single deletion of the *PKH1* or *PKH3* genes does not significantly modify the expression profiles of cells cultivated in optimal conditions (our unpublished results and [16, 17]). In fact, *pkh1* and *pkh3* single mutant cells have been included in the set of 784 non-responsive mutants because three or less significant mRNA expression changes were detected as a result of the single deletion of *PKH1* or *PKH3* [16]. Our aim is to discern the global transcriptional changes triggered by the lack of all of the three yeast Pkh proteins. For this purpose we used the tetO_7_-based system to produce a progressive depletion of Pkh by incubation of *pkh1 pkh3* double mutant cells in the presence of doxycycline for 8 and 24 h. We show that the levels of mRNA involved in the glycogen accumulation and in the responses to heat and oxidative stresses, as well as the unfolded protein response, are increased, whereas the levels of those related with ion transport are decreased. In addition, we validated the transcriptional data with experimental information using gene reporters and cell sensitivity to several environmental stresses.

Finally, we determined the need of Pkh for the proper Hsf1 and Msn2/4-driven transcriptional response to heat stress, but not for the down-regulation of genes coding for ribosomal proteins under these conditions.

## Results

### Depletion of Pkh proteins causes specific transcriptional changes

Previous large-scale studies have identified minor changes in the transcriptional patterns by the single deletion of *PKH1* or *PKH3* [16, 17]. This is not unexpected, reinforcing the notion that Pkh proteins have redundant functions and that the lack of one Pkh can be probably compensated by the presence of any of the other two Pkh proteins. Now, we have constructed the SDP8 yeast strain (Table 1) in which *PKH1* and *PKH3* genes are deleted and the quantity of Pkh2 can be progressively decreased by incubation in the presence of doxycycline [15]. According to quantitative RT-PCR experiments, incubation of the cells containing the tetO_7_-*PKH2* construct for 24 h in the presence of 100 μg/ml doxycycline reduced more than 200-fold the expression of *PKH2* (not shown). At this point most of the cells are still viable [15], probably because enough amounts of Pkh2 protein could still be present in the cell (the estimated half-life of Pkh2 is longer than 20 h). In order to gain insight into the cellular roles of the yeast PDK1 kinases, we decided to use this cellular system to identify the transcriptomic changes triggered by progressive depletion of Pkh proteins. To this end, we compared the expression pattern of the strain SDP8 with that of parental wild-type CML476 cells, grown both in the presence of 100 μg/ml doxycycline for 8 or 24 h. The expression patterns of antibiotic-treated versus untreated SDP8 cells were not compared because, in contrast to the previously published data [18], we have detected a small but significant number of transcriptional changes induced by doxycycline (our unpublished results).

**Table 1:** Yeast strains used in this study

Strain	Genotype	*PKH* gene(s) expressed in the presence of doxycycline	Source
CML476	*MATa ura3–52 leu*Δ*1 his3*Δ*200 GAL2* CMVp(tetR’-SSN6)::*LEU2 trp1::tTA*	*PKH1*, *PKH2*, *PKH3*	[55]
MB002	*MATa* CML476 KanMX4-(tetO_7_):*PKH2*	*PKH1*, *PKH3*	[15]
MB005	*MATa* CML476 KanMX4-(tetO_7_):*PKH2 pkh1*::*HIS3*	*PKH3*	[15]
SDP7	*MATa* CML476 KanMX4-(tetO_7_):*PKH2 pkh3*::*nat1*	*PKH1*	[15]
SDP8	*MATa* CML476 KanMX4-(tetO_7_):*PKH2 pkh1*::*HIS3 pkh3*::*nat1*	-	[15]

**Fig. 1:**
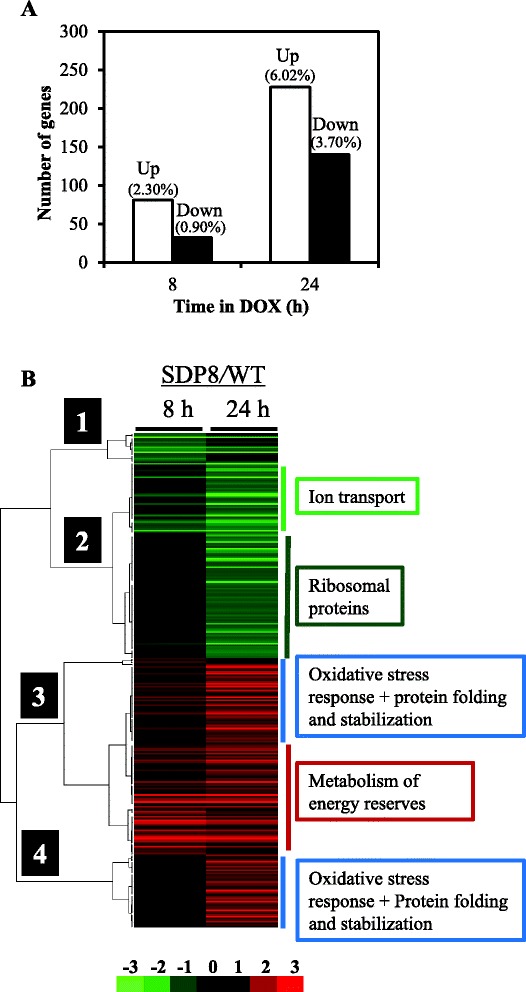
Gene expression changes triggered by gradual depletion of Pkh. **a** Number of genes found up-(empty bars) and down-regulated (filled bars) in SDP8 versus WT cells after 8 and 24 h of treatment with 100 μg/ml doxycycline. The percentage respect the total number of genes with valid expression value data are denoted in brackets. **b** Cluster analysis of the expression profiles caused by depletion of Pkh. Genes whose expression was found changed after treatment of SDP8 cells with doxycycline (either 8 or 24 h) when compared to wild-type cells under the same conditions were hierarchically clustered (complete linkage clustering, uncentered correlation) by means of the Gene Cluster (v. 2.11) software [72] and visualized with Java TreeView (v 3.0) [73]. Relevant functional categories of genes in each cluster are denoted

Our data indicated that incubation of SDP8 cells with doxycycline for 8 h changed the expression of 113 genes (3.1 % of the genes with valid data). Eighty-one were found up-regulated and 32 down-regulated (Fig. 1a and Additional file 1: Table S1). A longer incubation time in the presence of the antibiotic (24 h) increased the number of expression changes, affecting 9.7 % of the analyzed genes (228 and 140 up- and down-regulated, respectively). The expression patterns for the total number of genes whose expression was considered changed at any time-point are shown in the Fig. 1b. As hypothesized, deletion of *PKH1* and *PKH3* and progressive depletion of Pkh2 had more important transcriptional effects that deletion of any single *PKH* gene.

### Depletion of Pkh alters mRNA levels of genes required for glucose storage, ion transport and ribosomal proteins

**Fig. 2:**
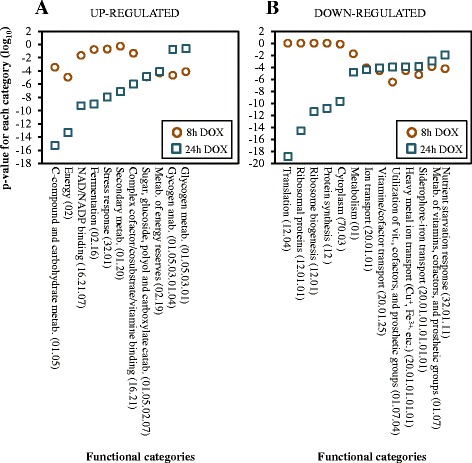
Relevant functional categories of the genes whose expression was affected by the progressive depletion of Pkh. **a** A set of the more represented functional categories, according to the MIPS FunCatDB [69], of the genes found up-regulated by incubation of SDP8 cells with doxycycline for 24 h is shown. The *p*-values for each category at both 8 and 24 h of treatment of SDP8 cells with doxycycline (circles and squared, respectively) are represented. **b** A set of the more represented functional categories, according to the MIPS FunCatDB, of the genes found down-regulated by incubation of SDP8 cells with doxycycline for 24 h is shown. The *p*-values for each category at 8 and 24 h of treatment of SDP8 cells with doxycycline (circles and squared, respectively) are represented

Functional analysis of the set of 81 genes found up-regulated after 8 h of incubation of SDP8 cells with doxycycline revealed an excess of genes involved in the energetic metabolism (19 genes; *p*-value: 1.09e-05) and in the metabolism of energy reserves (7 genes; *p*-value: 7.77e-05) (Fig. 2a). The mRNAs corresponding to genes coding for the high affinity glucose and maltose transporters (*HXT2*, *HXT4*, *HXT7* and *MAL31*) typically induced by glucose limitation, were also found among the most abundant (Additional file 1: Table S1). Incubation of SDP8 cells in the presence of doxycycline for 24 h resulted in the up-regulation of genes involved in carbohydrate metabolism (72 genes; *p*-value: 5.09e-16), energy (54 genes; *p*-value: 4.52e-14), fermentation (15 genes; *p*-value: 9.41e-10) and stress response (59 genes; *p*-value: 1.09e-08) among others (Fig. 2b).

**Fig. 3:**
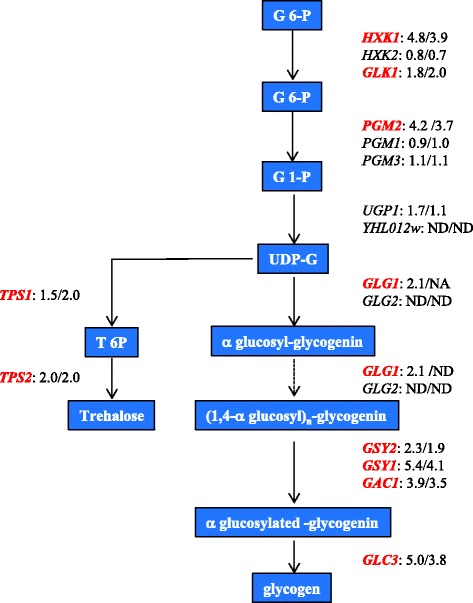
Depletion of Pkh affects glycogen metabolism. The complete glycogen and trehalose biosynthesis pathways, based on Yeast Biochemical Pathway Database (YeastCyc) at the SGD [74]. Numbers indicate the –fold induction of the specified genes in SDP8 when incubated in the presence of 100 μg/ml doxycycline for 8 and 24 h when compared to CML476 wild-type cells under the same treatment. Genes considered up-regulated are denoted in red. ND: no valid data were obtained

Most of the genes involved in glycogen biosynthesis have been found up-regulated when SDP8 cells were incubated in the presence of doxycycline for 8 h (Fig. 3). *GAC1*, which encodes for the regulatory subunit of Glc7, that tethers Glc7 to the glycogen synthase [19], is among these genes (Fig. 3). Expression of genes involved in the glycogen metabolism normally increases when cells approach the stationary phase in a PKA-dependent manner, involving the transcription factors Msn2 and Msn4 [20]. Glycogen accumulation also plays an important role in response to several stresses [21]. The mRNA levels of *TPS1* and *TPS2*, required for the synthesis of trehalose, were also found increased (Fig. 3). Trehalose is a reserve carbohydrate which is proposed to function as stress protectant that has been involved in stress responses by stabilizing proteins and membranes. Although the cellular levels of trehalose and glycogen are not directly related to the expression levels of the trehalose and glycogen synthase genes, high expression of those genes have been reported in cells under several stressful conditions including, but no limited to, heat shock, oxidative stress, alkaline pH and high osmolarity, [22–24].

**Fig. 4:**
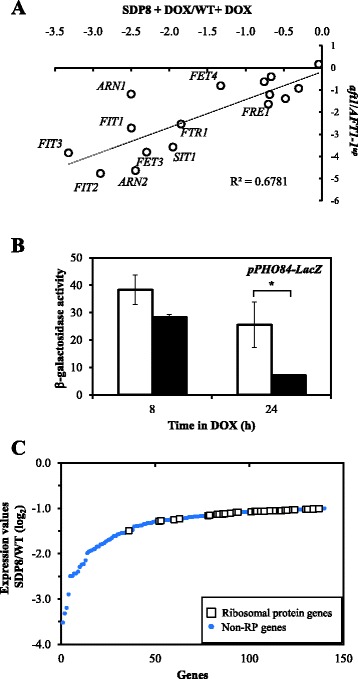
Depletion of Pkh causes down-regulation of genes involved in ion transport. **a** Aft1 dependence of *S. cerevisiae* iron transport genes and their down-regulation by depletion of Pkh. Comparison of the transcriptional changes, in log_2_, triggered by treatment of SDP8 cells with 100 μg/ml doxycycline for 24 h to CML476 when compared to wild-type cells under the same treatment (*x* axis) with data obtained when the expression pattern of *aft1* mutant cells was compared to that of a wild-type isogenic strain carrying the constitutively activated *AFT1*-*1* ^up^ allele [26]. **b** β-Galactosidase activity of cells containing the *PHO84*-LacZ reporter gene. SDP8 cells transformed with the plasmid *PHO84*-LacZ and liquid cultures of the resulting strain were treated with doxycycline (100 μg/ml) or the vehicle for 8 or 24 h, as specified in the Methods section. β-Galactosidase activity was measured in non-treated (empty bars) or treated (filled bars) cells. Data are mean ± SEM from three independent clones. Treated cells were compared with untreated cells at each time point using un-paired t-test (n = 3; one-tailed *P* value; *P < 0.05). **c** The 140 genes down-regulated by incubation of SDP8 cells with doxycycline for 24 h have been ordered according to their expression values when compared to the expression in wild-type cells under the same conditions. Genes coding for ribosomal proteins are denoted by empty squares

Analysis of the 32 down-regulated genes in SDP8 cells treated with doxycycline for 8 h (Additional file 2: Table S2) revealed that the category of iron transport (such as *SIT1*, *ARN1*, *ARN2*, *FRE1*, *FIT2*, *FIT3* and *PHO84* among others; *p*-value: 7.94e-05) were over-represented (Fig. 2b). This category had similar relevance when SDP8 cells were treated for 24 h with the antibiotic (*p*-value: 3.77e-05). These expression patterns recall the transcriptional changes described under iron-surplus conditions, controlled by the Aft1and Aft2 transcription factors [25]. In fact, there is a good correlation (R^2^ = 0.68) between the expression changes of the genes down-regulated by incubation of SDP8 cells with doxycycline for 24 h and the set of Aft1-dependent iron transport genes [26] (Fig. 4a).

The expression of genes coding for the high affinity inorganic phosphate transport system (*PHO84*), and the secreted acid phosphatases (*PHO5*, *PHO11*, *PHO12* and *PHO3*) that mediate the response to phosphate acquisition, were also found down-regulated at both, 8 and 24 h of SDP8 cells treatment with doxycycline (Additional file 2: Table S2). We confirmed the decreased expression levels of the high-affinity inorganic phosphate transporter gene *PHO84* by using the LacZ reporter gene fused to its promoter when SDP8 cells were incubated with doxycycline for 24 h (Fig. 4b).

When the set of 140 genes down-regulated by incubation of SDP8 cells with doxycycline for 24 h were considered, it is noticeable that an excess of genes involved in the translation process was manifested (36 genes; *p*-value: 1.26e-19; Fig. 2b and Additional file 2: Table S2). Among them, the sub-category of genes coding for ribosomal proteins was the more relevant (31 genes; *p*-value: 2.69e-15). Genes coding for ribosomal proteins were only moderately down-regulated as a consequence of the long-term depletion of Pkh, as shown in Fig. 4c, and their RNA levels were not affected when SDP8 cells were treated with doxycycline for 8 h.

Our results provide evidence for the existence of a set of genes whose mRNA levels changed just 8 h after starting the depletion of Pkh. Expression of other genes was not altered at this time-point and only changed after prolonged incubation with doxycycline, which could be an indirect consequence of early cellular events caused by depletion of Pkh.

### Progressive depletion of Pkh increases the transcription of genes required for fermentation and stress responses

Depletion of Pkh led to progressive accumulation of mRNA of those genes involved in carbohydrate metabolism. Among the 81 and 228 genes found up-regulated after 8 and 24 h of treatment of SDP8 cells with doxycycline, 21 and 72, respectively, belong to this category (*p*-values: 3.49e-04 and 5.09e-16, respectively; Fig. 2a). Careful analysis indicates that this response was not identical for all sub-categories of carbohydrate metabolism. Although the relevance of the genes included in the sub-category of sugar, glucoside, polyol and carboxylate catabolism was similar at both time-points (1.20e-05 and 1.30e-05, Fig. 2a), genes involved in the fermentation sub-category were preferentially up-regulated after long-term treatment of SDP8 cells. In fact, only two genes of this sub-category were found up-regulated after 8 h of doxycycline treatment (*ALD4* and *BDH2*; *p*-value: 1.68e-01) in contrast to 15 genes found after treatment for 24 h, mainly involved in redox processes (*AAD3*, *AAD6*, *AAD14*, *AAD15*, *AAD16*, *ACS1*, *ALD2*, *ALD3*, *ALD4*, *ALD6*, *ARO10*, *BDH2*, *DLD1*, *NDE2* and *NGL3*; *p*-value: 9.41e-10).

In SDP8 cells incubated for 8 h with doxycycline we have found increased the mRNA levels of a set of 13 genes involved in the stress response but not specifically implicated in any particular sub-category of stress (*AGP2*, *CYC7*, *FMP43*, *GAC1*, *GAD1*, *GCY1*, *HSP26*, *MGA1*, *MSN4*, *STF2*, *TMA10*, *PRB1* and *YFL054C*; *p*-value: 1.96e-01). Much more relevant, however, was the set of genes up-regulated when the same cells were incubated for 24 h with the antibiotic (59 genes; *p*-value: 1.09e-08) (Fig. 2a). Among these genes the more significant sub-categories represented, shown in Table 1, are: i) the unfolded protein response (14 genes; *p*-value: 4.02e-05) containing genes coding for proteins with chaperone/co-chaperon activities (*APJ1*, *CPR6*, *HSP26*, *HSP33*, *HSP42*, *HSP78*, *HSP82*, *MDJ1* among other; ii) oxidative stress response (11 genes; *p*-value: 4.58e-04) including *CTA1*, coding for catalase A, the cytoplasmic peroxiredoxin *TSA1*, the mitochondrial thioredoxin peroxidases *PRX1*, the mitochondrial superoxide dismutase *SOD2*, the genes involved in the 4-aminobutyrate metabolism *GAD1* and *UGA2* and iii) heat-shock response (8 genes; *p*-value: 9.80e-04). Changes in expression for several of the genes detected in our microarrays analysis were also verified by semiquantitative RT-PCR (Additional file 3: Figure S1).

To validate the significance of the described transcriptional changes, a publication enrichment analysis with the set of 228 genes found up-regulated after treating SDP8 cells with doxycycline for 24 h was performed. The results indicated that 45 of these genes have been previously found up-regulated in response to mild heat shock (*p*-value: 6.13e-23) [27] among other stress conditions that also induced comparable stress response [28]. However, the most similar expression pattern found was that triggered by treatment of wild-type cells with 0.6 M NaCl for 45 min [29]. From the set of 136 genes induced after the salt stress, 54 genes (mostly involved in oxidative stress response and metabolism of carbohydrates such as the pentose-phosphate pathway), were also up-regulated in SDP8 cells treated with doxycycline for 24 h (Additional file 1: Table S1).

### Depletion of Pkh mimics a transcriptional response triggered by environmental stressors

Our data suggest that depletion of Pkh triggers, directly or indirectly, a common yeast response to different stresses. Examination of the transcriptional response to many types of environmental stresses allowed the identification of a gene expression program known as environmental stress response (ESR) [22]. A total number of 867 ESR genes were reported, being the expression of 585 and 282 genes, respectively, up- or down-regulated by environmental stresses [22]. From these subsets, 86 and 56 have been found to be also up- and down-regulated by depletion of Pkh (Additional file 1: Table S1 and Additional file 2: Table S2), which represent an enrichment of 5- and 2.5-fold over the expected number in case of independent events. Thus, the transcriptional response triggered by depletion of Pkh correlated to that of the ESR program.

Several of the ESR genes have paralogs in the yeast genome whose expression does not respond to ESR [30]. In order to know if the up-regulated genes correspond or not to the ESR genes we calculated expression changes for the set of ESR and non-ESR paralogs. Our analysis indicated that the averages of the expression changes (in log_2_) observed for the ESR genes were 0.72 and 1.01 for the cells treated with doxycycline for 8 and 24 h, respectively. By contrast, the averages for the non-ESR paralogs were −0.05 and 0.03 respectively. This indicates that Pkh depletion causes the specific up-regulation of ESR genes but, in most cases, it does not increase the expression of their non-ESR paralogs (Additional file 4: Table S3). For example, typical ESR genes involved in the metabolisms of carbohydrates such as the hexokinase *HXK1*, the glucokinase *GLK1* or the phosphoglucomutase *PGM2*, were up-regulated when Pkh was depleted but the expression of their non-ESR paralogs *HXK2*, *EMI2* and *PGM1* was not increased. These results suggest that, directly or indirectly, progressive depletion of Pkh preferently triggers the expression of ESR genes.

**Fig. 5:**
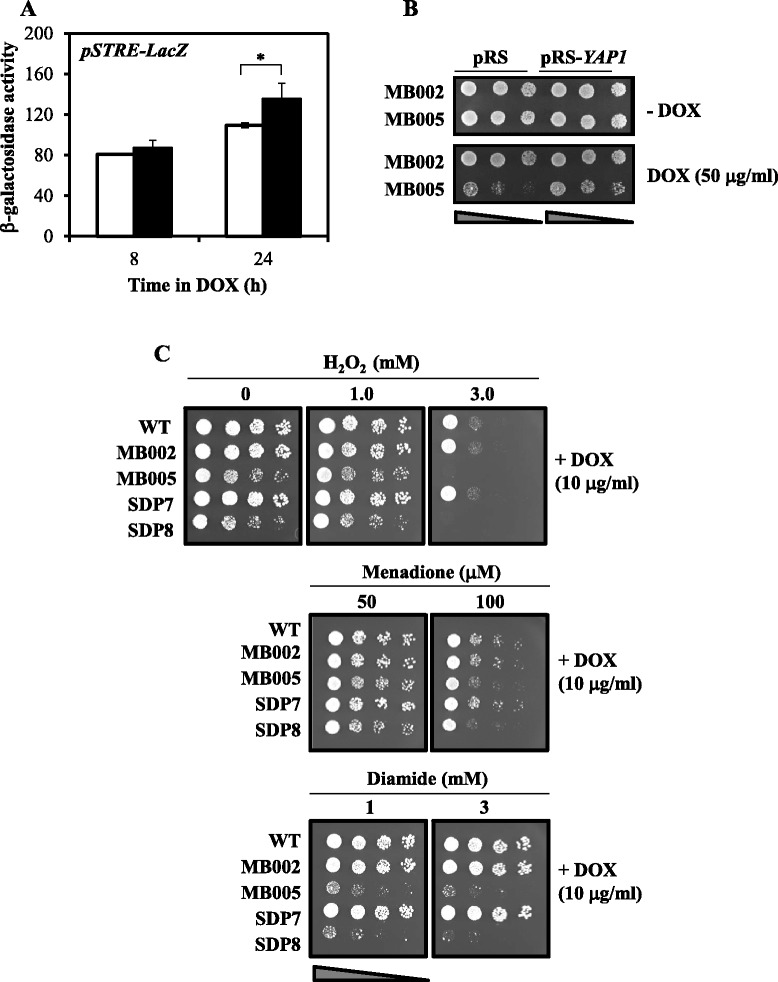
Cells lacking Pkh are hypersensitive to oxidative stress. **a** β-Galactosidase activity of cells containing the *STRE-LacZ* reporter gene. SDP8 were transformed with the pGM18/17 plasmid, which was previously linearized at the *URA3* gene marker by digestion with NotI, and treated as described for the Fig. 4a. β-Galactosidase activity was measured in non-treated (empty bars) or treated (filled bars) cells. Data are mean ± SEM from three independent clones. Treated cells were compared with untreated cells at each time point using un-paired t-test (n = 3; one-tailed *P* value; *P < 0.05). **b** Effect of partial depletion of Pkh on the sensitivity to oxidative agents. Wild type (WT) CML476 cells and the indicated mutants were grown, and four different (1:5) dilutions of the cultures containing the same number of cells were spotted on YPD plates containing 10 μg/ml doxycycline and the indicated concentrations of H_2_O_2_, menadione and diamide. Growth was monitored after 3 days. **c** Overexpression of *YAP1* partially rescues the lethal effect of depletion of Pkh. MB002 and MB005 cells were transformed with the empty centromeric pRS316 or the same plasmid containing the promoter and coding region of *YAP1*. Serial dilutions of liquid cultures were spotted on YPD plates containing 50 μg/ml doxycycline (DOX) or the vehicle. Cell growth was recorded after 3 days

Several transcription factors are involved in the transcriptional changes caused by specific stress conditions, being Msn2 and Msn4 major players in this response. These transcription factors recognize and bind the Stress Response Element (STRE), sequences found in most of the promoters of the stress-responsive genes including glycogen and trehalose synthesis [30, 31]. To test if the transcription factors Msn2/Msn4 are activated during the stress-related transcriptional response found in Pkh-depleted cells we used the STRE sequence fused to the lacZ reporter gene. As shown in Fig. 5a, activity of the β–galactosidase gene product driven by the STRE was slightly higher after depletion of Pkh. From these results we can conclude that depletion of Pkh triggers a stress-like transcriptional response that is, at least in part, mediated by the Msn2/Msn4 transcription factors.

### Lethality caused by depletion of Pkh could be partially suppressed by overexpression of *YAP1*

Pkh-depleted cells transcriptionally respond to stress. It is reasonable to think that the observed response to oxidative stress could be the consequence of a situation of cellular oxidative stress directly or indirectly caused by depletion of Pkh. We next tested the tolerance to oxidative stress of SDP8 cells incubated in the presence of low (non-lethal) doses of doxycycline. Our results indicate that partial depletion of Pkh made cells hypersensitive to the oxidative agent hydrogen peroxide and to the thiol oxidant diamide, but cells were less affected by the superoxide generator menadione (Fig. 5b). These results could be explained assuming that Pkh-deficient cells suffer of oxidative stress, which can explain the increased levels of reactive oxygen species (ROS) previously observed in these cells [15]. To test if the high levels of ROS could be the cause of Pkh-deficient cells lethality, we overexpressed the transcription factor Yap1, which is specifically required for oxidative stress tolerance [32] in MB005 cells (Table 1). As observed in Fig. 5c, expression of *YAP1* from a centromeric plasmid partially rescued the lethality provoked by the combined deletion of *PKH1* and depletion of Pkh2 induced by incubation of MB005 cells with 50 μg/ml of doxycycline.

This result shows that the lethality driven by depletion of Pkh is, at least in part, due to the cellular oxidative stress since it can be partially alleviated by overexpression of *YAP1*. The transcriptional changes observed in Pkh-depleted cells dedicated to respond to the oxidative stress are probably insufficient to cope with stress and cells become hypersensitive to environmental oxidative stresses.

### Pkh-depleted cells are hypersensitive to UPR-inducers and toxic ions

The accumulation of unfolded or misfolded proteins in the endoplasmic reticulum triggers the conserved unfolded protein response (UPR). The objective of the UPR is to reduce the translation and, simultaneously, to initiate a signaling pathway that leads to activation of Hac1. Hac1 is a key transcription factor that increases the transcription of genes involved in proper protein folding (see [33] for a recent review). Depletion of Pkh1 caused the up-regulation of a significant number of genes involved in the UPR (Table 2). Comparison of the full expression pattern of cells treated with the tunicamycin [34], an inhibitor of the protein glycosylation that triggers the UPR, with that of Pkh-depleted cells evidences that the similarities also extended to the down-regulated genes. In fact, the number of genes up- or down-regulated by treatment with tunicamycin and by depletion of Pkh, was higher than the expected in case of independent events (2.1- and 7.2- fold, respectively; Additional file 1: Table S1 and Additional file 2: Table S2).

**Table 2:** Stress response-related genes up-regulated by progressive depletion of Pkh

Gene	UPR	Oxidative	Heat shock	Description
*APJ1*	X		X	Chaperone of the HSP40 family.
*CPR6*	X		X	Peptidyl-prolyl cis-trans isomerase (cyclophilin).
*CTA1*		X		Catalase A.
*GAC1*			X	Regulatory subunit for Glc7p type-1 protein phosphatase (PP1).
*GAD1*		X		Glutamate decarboxylase.
*GRE1*		X	X	Hydrophilin essential in desiccation-rehydration process.
*GRE3*			X	Aldose reductase involved .
*HSP12*		X	X	Plasma membrane protein that protects membranes from desiccation.
*HSP26*	X^a^			Small heat shock protein (sHSP) with chaperone activity.
*HSP31*	X			Methylglyoxalase that converts methylglyoxal to D-lactate.
*HSP33*	X			Possible chaperone and cysteine protease.
*HSP42*	X^a^			Small heat shock protein (sHSP) with chaperone activity.
*HSP78*	X			Oligomeric mitochondrial matrix chaperone.
*HSP82*	X^a^			Hsp90 chaperone.
*HSP104*	X^a^		X	Disaggregase.
*MCR1*		X		Mitochondrial NADH-cytochrome b5 reductase.
*MDJ1*	X			Co-chaperone that stimulates HSP70 protein Ssc1p ATPase activity.
*ORM2*	X			Protein that mediates sphingolipid homeostasis.
*OXR1*		X		Protein of unknown function required for oxidative damage resistance.
*PRX1*		X		Mitochondrial peroxiredoxin with thioredoxin peroxidase activity.
*SNQ2*		X		Plasma membrane ATP-binding cassette (ABC) transporter.
*SOD2*		X		Mitochondrial manganese superoxide dismutase.
*SSA3*	X			ATPase involved in protein folding and the response to stress.
*SSA4*	X_a_			Heat shock protein that is highly induced upon stress.
*SSE2*	X^a^		X	Member of the heat shock protein 70 (HSP70) family.
*TSA2*		X		Stress inducible cytoplasmic thioredoxin peroxidase.
*UBC5*	X^a^			Ubiquitin-conjugating enzyme.
*UGA2*		X		Succinate semialdehyde dehydrogenase.

Reported involvement in the UPR and responses to oxidative and heat stresses of the set of genes up-regulated by depletion of Pkh, according to the FunCatDB

UPR: 32.01.07 (Unfolded protein response); oxidative: 32.01.01 (oxidative stress response); Heat shock: 32.01.05 (heat shock response). ^a^the up-regulation of these genes has been described as independent of Hac1 [75]

**Fig. 6:**
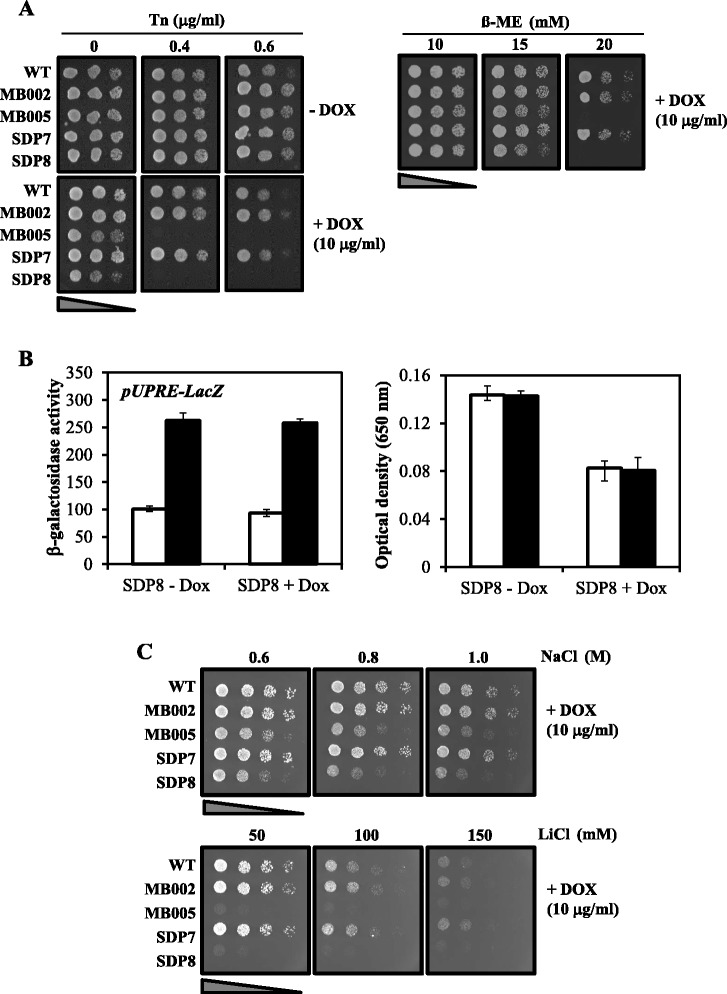
Cells lacking Pkh are sensitive to UPR inducers and ionic stress. **a** Effect of partial depletion of Pkh on the sensitivity to the UPR inducers tunicamycin and β-mercaptoethanol. Cultures of CML476 cells (WT) and its specified derivatives were serially diluted and spotted onto YPD plates containing 10 μg/ml doxycycline (DOX), or the vehicle, and the indicated concentrations of tunicamycin (Tn) or β-mercaptoethanol (β-ME). Control plate in the absence of doxycycline of cells incubated with β-ME is shown in the Fig. 5b. Cell growth was recorded after 3 days. **b** Left panel. β-Galactosidase activity of SDP8 cells containing the *UPRE-LacZ* reporter gene. SDP8 were transformed with the pMCZ-Y plasmid and incubated in medium without uracil in the presence of 10 μg/ml of doxycycline (“SDP8-Dox”) or the vehicle (“SDP8 + Dox”) for a total time of 24 h. Six h before the treatment with tunicamycin, new cells cultures at OD_660_ ~ 0.05 were prepared containing fresh doxycycline. Finally, cells were resuspended in medium containing fresh doxycycline (or the vehicle) and 2.5 μg/ml tunicamycin (or DMSO). β-Galactosidase activity was measured for cells non-treated (empty bars) or treated with tunicamycin (filled bars). Data are mean ± SEM from six independent clones. Right panel: Representation of the cell density of the cultures used in the left panel obtained by measuring their absorbance at 650 nm in a microplate reader, after the treatment of SDP8 cells with tunicamycin (filled bars) or with the vehicle (empty bars). Data are mean ± SEM from six independent clones. **c** Effect of partial depletion of Pkh on the salts sensitivity. Four dilutions (1:5) of cells from the strains specified in **a** were spotted onto YPD plates containing 10 μg/ml doxycycline (DOX) and the indicated concentrations of NaCl or LiCl. Control plate for the absence of doxycycline is shown in the Fig. 5b. Cell growth was recorded after 3 days

To further study the possible connection between depletion of Pkh and the UPR we tested the sensitivity to tunicamycin of cells containing low levels of Pkh. Incubation of SDP8 or MB005 cells in the presence of low doses of doxycycline greatly increased the hypersensitivity to tunicamycin when compared to wild-type cells (Fig. 6a). Pkh-deficient cells were also hypersensitive to β-mercaptoethanol, another agent that activates the UPR, although in a less-specific manner (Fig. 6a). These results indicate that partial depletion of Pkh somehow mimics an ER-stress-induced transcriptional response that could be caused by the progressive accumulation of unfolded proteins or by interfering with the UPR signaling pathway, among other options. Partial depletion of Pkh, however, did not change the basal transcriptional activity driven by the UPRE, although it did increase the cell duplication time (Fig. 6b). Furthermore, decreased levels of Pkh, did not interfere with the normal response triggered by tunicamycin (Fig. 6b).

The transcriptional response of Pkh-depleted cells was also to some extent similar to that induced by saline stresses [29]. We then investigated the effect of low levels of Pkh on salt tolerance. As shown in the Fig. 6c, SDP8 and MB005 cells treated with low doses of doxycycline were hypersensitive to sodium and lithium toxic ions, suggesting that cells expressing low doses of Pkh could be already challenged by an ionic stress, and cannot handle additional environmental ionic stress.

### Progressive depletion of Pkh affects the expression of specific transcription factors

From the present data we concluded that depletion of Pkh triggers a transcriptional response addressed to cope with several types of stress. In order to know which transcription factors are mainly affected by the lack of Pkh, the sets of 82 and 228 genes whose expression was increased by incubation of SDP8 cells with doxycycline for 8 and 24 h, respectively were first analyzed. Our results showed that the transcription factors involved in stress responses, such as Msn2, Msn4, Adr1, Hsf1, etc., were the most affected (Additional file 5: Figure S2). Table 3 displays the complete list of relevant transcription factor affected by the progressive depletion of Pkh. Overall, there is no much difference between the transcription factors affected by incubation of SDP8 cells with doxycycline for 8 h and those observed after 24 h. However, when the same analysis was performed only with down-regulated genes, there were different relevant transcription factors affected by 8 and 24 h of doxycycline treatment. Thus, transcription factors such as Srb5, Ask10 or Stb1 (*p*-value from 6.2e-08 to 3.4e-06) were most significantly altered after 8 h incubation with doxycycline, controlling only a few genes (*PHO3*, *PHO5*, *SIT1*, *ARN1*, *ARN2*, *PHO12*, *PHO84*, *FIT2* and *FIT3*, in the case of Stb1). Longer incubation of SDP8 cells with doxycycline completely changed the transcription factors involved, being more relevant those controlling the transcription of ribosomal proteins (Ifh1: 45 genes; Fhl1: 62 genes), Spt23 (88 genes), Hmo1 (61 genes) and Rap1 (103 genes).

**Table 3:** Transcription factors mainly affected by progressive depletion of Pkh

	8 h of doxycycline		24 h of doxycycline	
	Number of		Number of	
T.F.	Target genes	*p*-value	Target genes	*p*-value
Msn2	72	1.00E-15	183	0
Msn4	58	1.52E-11	152	0
Spt23	51	2.77E-13	148	0
Rlm1	38	0	94	0
Mga2	37	2.38E-11	103	0
Adr1	37	2.00E-15	86	0
Crz1	28	4.50E-14	68	0
Rgm1	28	0	59	0
Wtm2	19	1.00E-15	31	0
Ric1	18	9.08E-05	64	0
Tog1	17	1.28E-11	35	0
Com2	17	5.04E-13	31	0
Gis1	12	2.58E-04	52	0
Swi4	31	6.18E-08	81	1.00E-15
Hot1	12	2.10E-12	21	1.00E-15
Hsf1	28	1.32E-02	108	3.00E-15
Flo8	26	3.44E-10	56	5.90E-14
Cbf1	40	5.62E-09	97	6.70E-14
Rpi1	15	6.54E-11	28	1.00E-13
Sko1	31	1.78E-09	70	2.49E-13
Pdr1	39	3.78E-09	90	2.78E-12
Cin5	48	2.47E-08	118	3.72E-12
Gat4	6	1.19E-02	29	7.35E-12
Yap6	29	1.12E-07	65	6.90E-10

The set of genes up-regulated by incubation of SDP8 cells with 100 μg/ml doxycycline for 8 or 24 h are ranked by transcription factor, according to the *Rank by TF* application of the Yeastract platform and using the DNA binding plus expression evidence and TF acting as activator. The number of genes whose expression is controlled by each transcription factor is shown. A score for each transcription factor is given by a *p*-value that denotes the overrepresentation of the given transcription factor targeted genes in the list of up-regulated genes relative to the regulations of that transcription factor targeted genes in the whole Yeastract database

### Pkh-depleted cells displayed an attenuated transcriptional response to heat shock

**Fig. 7:**
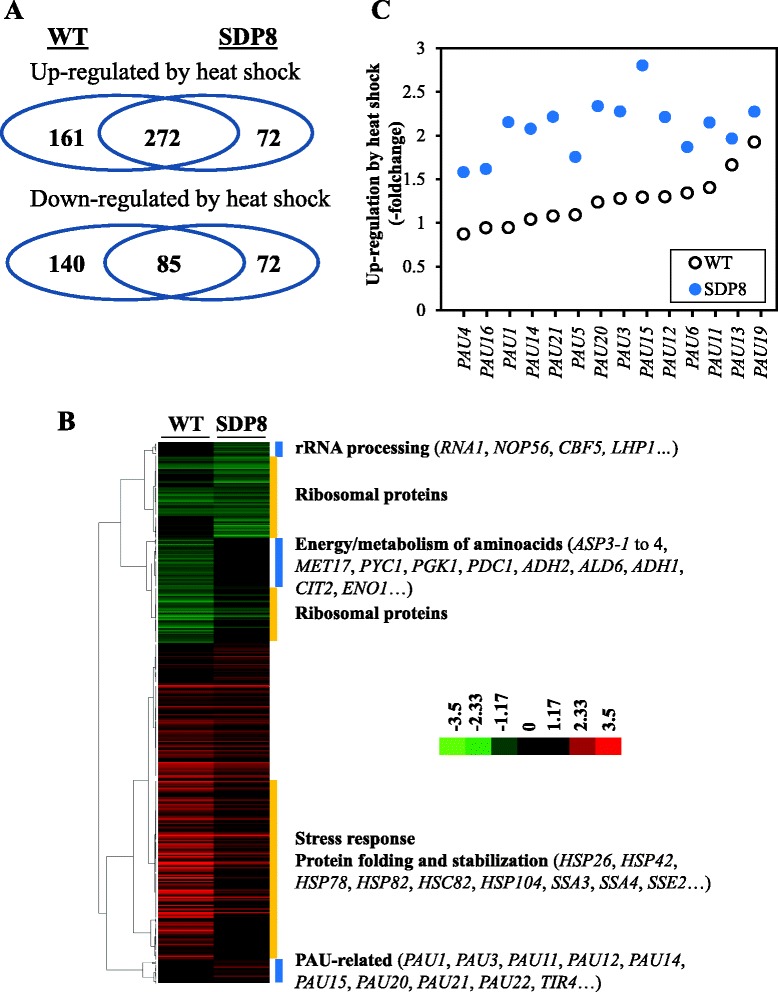
Transcriptional response of Pkh-depleted cells to heat shock. **a** Venn diagrams of the number of genes with considered up-regulated (upper panel) and down-regulated (lower panel) mRNA expression under heat sock stress in the wild-type CML476 (WT) and SDP8 strains. **b** The expression patterns of the wild-type CML476 (WT) and SDP8 cells treated with doxycycline for 24 h and incubated at 40 °C for 40 min were compared with those of the same cells in the absence of the heat stress. A set of 803 genes with data in both strains that are differentially expressed at least in one of the strains were hierarchically clustered (complete linkage clustering, uncentered correlation) using the Gene Cluster software (v. 2.11) and visualized with Java TreeView (v 3.0). The relevant functional categories of genes in some clusters are denoted. **c** The mRNA levels (in –fold change) determined by DNA microarrays for the PAU family of genes after heat shock are represented for the CML476 (WT, empty circles) and SDP8 (filled circles) strains

We have shown that depletion of Pkh triggers a heat stress-like transcriptional response. It has been described that Pkh activity is regulated by sphingolipids and these lipids play an important role in the cellular response to a series of stresses, including heat stress [35]. For this reason we next characterized the transcriptional responses of wild-type and Pkh-depleted cells to heat stress. Incubation of doxycycline-treated CML476 wild-type cells at 40 °C for 40 min caused the up-regulation of 11.6 % of genes with valid data, mainly those involved in the stress response and protein folding and stabilization. We also observed that 6.0 % of the genes, mostly coding for ribosomal proteins, were down-regulated. When doxycycline-treated SDP8 cells were heat stressed in the same way, only 9.5 % and 4.1 % were found up- and down-regulated, respectively, suggesting the attenuation of the transcriptional response (Additional file 6: Figure S3), although the functional categories were the same than those of stressed wild-type cells. Similar results were obtained when only genes with valid data for the two strains were considered (Fig. 7a and Additional file 7: Figure S4), indicating a general attenuation of the transcriptional response to heat stress in Pkh-depleted cells. We next identified the genes whose expression changes under heat stress were dependent on the presence of Pkh. We have found that the expression changes of 271 genes (41.2 %) were somehow dependent on the presence of Pkh: 193 up-regulated (44.6 %, Additional file 8: Table S4) and 78 were down-regulated (34.7 %, Additional file 9: Table S5).

It is worth mentioning that genes involved in the response to stress and in protein folding and stabilization were up-regulated preferably in a Pkh-dependent manner (Additional file 8: Table S4). Among the genes down-regulated in a Pkh-dependent manner, those involved in the metabolism of the aspartate family of amino acids were found overrepresented. The regulation of most of the genes encoding ribosomal proteins was essentially independent on Pkh (Fig. 7b and Additional file 10: Table S6). We have also found a set of genes whose expression levels under heat shock changed only in Pkh-depleted but not in wild-type cells. For example, an excess of the seripauperin multigene family (*PAU* genes) encoded mainly by subtelomeric regions of the genome were found up-regulated in response to heat shock only in doxycycline-treated SDP8 cells (Fig. 7b and c). It has been suggested that this family of proteins might possess specific roles in the cell adaptation to certain environmental stresses [36]. Similarly, the mRNA levels of a set of genes involved in the maturation of rRNA were down-regulated after heat shock in SDP8 but not in wild-type cells (Fig. 7b). These results indicate that there is a relevant set of genes whose expression changes under heat-shock conditions depends on the presence of Pkh.

### The full transcriptional response to heat shock requires Pkh proteins

The above data indicate that depletion of Pkh influences, directly or indirectly, the transcriptional response elicited by heat stress, affecting genes involved in the stress response and the metabolism of energy reserves and amino acids. It is plausible to think that the already elevated levels of mRNA involved in stress responses found in Pkh-depleted cells could prevent further up-regulation of this set of genes in response to heat shock. In fact, we have found a high degree of correlation when the set of genes up-regulated by the depletion of Pkh were compared to those up-regulated by heat shock in wild-type cells (slope: 0.735). This argument, however, is not valid for the set of genes down-regulated by depletion of Pkh1 since there is no correlation at all with those down-regulated by heat shock (slope: −0.163). To test the above-mentioned hypothesis we analyzed the set of 193 genes whose up-regulated expression in wild-type cells by heat shock was classified as dependent on Pkh. The mRNA quantity of 52 of these genes was found already increased in Pkh-depleted cells, when SDP8 cells were incubated with doxycycline for 24 h (Additional file 6: Figure S3A). The low induction levels of this set of genes under heat shock conditions could be then explained by their high expression levels in Pkh-depleted cells under basal conditions. However, the expression levels of 91 genes, also up-regulated by heat shock in a Pkh-dependent manner, were not found increased under basal conditions in Pkh-depleted cells (Additional file 6: Figure S3B). We can consider that the lack of induction of this set of genes by heat shock in Pkh-depleted cells could be a consequence of Pkh depletion. Although genes involved in protein folding and stabilization and stress response are present in both subsets of genes, its relevance is higher in the subset of already up-regulated genes in Pkh-depleted cells. By contrast, we found higher proportion of genes involved in detoxification in the subset that were not found up-regulated by Pkh depletion (7 genes: *CUP1-1*, *BSD2*, *GRX6*, *GRX4*, *TPO1*, *TPO3* and *SGE1*; *p*-value: 8.09e-03) than in the subset of genes found up-regulated by the depletion of Pkh (2 genes: RTA1 and TPO4; *p*-value: 4.06e-01). From these analyses we can conclude that there is a set of genes whose up-regulation under heat shock stress is, directly or indirectly, dependent on the presence of normal levels of Pkh.

Therefore, the lower up-regulation detected for this set of genes in SDP8 cells subjected to heat stress is not due to the already elevated levels resulting from Pkh depletion in non-stressed conditions. On the contrary, the attenuated transcriptional response after heat shock of this set of genes could be considered the consequence of Pkh deficiency.

## Discussion

In this work we have contributed to the identification of new cellular roles of Pkh by describing the transcriptional patterns triggered by the absence of the Pkh proteins under various conditions. Pkh proteins provide an essential function in diverse yeast species by regulating an increasing number of cellular functions. Most of the studies on Pkh have been performed using a temperature-sensitive allele [9]. However, this strain does not allow the identification of the transcriptional response of Pkh-deficient cells in optimal growth conditions since a shift to a stressful temperature is required to reduce the cellular Pkh activity. We have previously described that Pkh-deficient cells are hypersensitive to heat stress [15]. For this reason we have developed a different approach based on a doxycycline-regulated promoter that allowed us to identify for the first time the transcriptional changes triggered by the progressive depletion of Pkh proteins in the yeast *S. cerevisiae*. It is worth mentioning that depletion of Pkh proteins by this strategy does not imply a stressful growth condition for the cells and that, in any case, we have compared the expression profiles of two strains cultivated in the presence of doxycycline. Furthermore, the use of the doxycycline-regulated promoter allowed us to identify the transcriptional response of Pkh-deficient cells to heat stress, an experiment that cannot be properly done by using a strain carrying a temperature-sensitive allele.

Until now, transcriptional changes caused only by the single deletion of *PKH1* or *PKH3* have been identified in large-scale studies [16, 17]. Using the published data, and according to our criteria, lack of *PKH1* triggers the up-regulation of two contiguous genes located in the chromosome IV, *BSC1* (YDL037C) and *PRM7* (YDL039C). *PHO84* was the only down-regulated gene in this mutant strain. In the same study lack of *PKH3* up-regulates the same genes described for the *pkh1* mutant cells plus YDL196W, and down-regulates *PHO84* and also *SPL2*. It is worth to note that the mRNA levels of genes like *FIT3*, *SPL2* and *FIT2* were found reduced in *pkh1* mutant cells (0.50, 0.57 and 0.60-fold, respectively) [16]. In another study lack of Pkh1 or Pkh3 had not relevant consequences on the expression profiles [17]. Our study demonstrates that expression changes caused by the double deletion of *PKH1* and *PKH3* and depletion of Pkh2 are reminiscent of those triggered in response to environmental stresses [37]. We show that the expression of genes involved in the metabolism of glycogen and trehalose as well as in stress responses were already elevated in the first time-point assessed, and increased moderately or did not increase along the rest of the experiment. This early transcriptional response, is probably a direct consequence of the decreased levels of Pkh and is comparable to that described for several environmental stresses, where the expression levels for genes involved in glycogen metabolism increases soon immediately after the stress and then the mRNA levels for these genes progressively decrease [38, 39]. Up-regulation of the genes required for high-affinity glucose transport and phosphorylation as well as for glycogen accumulation have also been found in heat-stressed cells [30]. Genes involved in the defense against oxidative stress and accumulation of unfolded protein increased their expression along the experiment, suggesting that depletion of Pkh could lead to cellular stress or, at least, trigger a response inducible by stress. In this agreement, we also observed a decrease in the quantity of mRNA coding for ribosomal proteins and other mRNA related to protein synthesis. Oxidative stress and stress driven by the accumulation of improperly folded or unfolded proteins are coupled since the former disrupts the proper disulfide formation thus inducing the UPR [40]. Then, the observed transcriptional response could be primarily driven by the oxidative stress. Oxidative stress can be triggered by several factors, including a malfunction of the pentose phosphate pathway, which function to regenerate NADPH for recycling the oxidized glutathione [41]. According to this, deletion of the gene coding for the glucose 6-phosphate dehydrogenase (*ZWF1*), enzyme of the first reaction of the pentose phosphate pathway, renders respiring cells with high levels of ROS [42]. We observed that lower than normal levels of Pkh does not affect neither the basal levels of transcription driven by the UPRE nor the activation of this pathway by UPR-inducers. Hypersensitivity to UPR-stressors of cells with low levels of Pkh could be explained by other reasons. Low Pkh levels might, for example, affect the recovery of the UPR after removal of the stress. A delay in the recovery of the UPR is the cause of the hypersensitivity to UPR stressors displayed by *reg1* mutant cells [43].

We also detected the short-term down-regulation of genes required for phosphate and iron uptake. It is known that members of the regulon PHO are down-regulated as soon as 15 min after the beginning of a heat stress [44]. Expression of the genes coding for the high-affinity phosphate transporters *PHO84* and *PHO89*, and the secreted acid phosphatases *PHO11*, *PHO12* and *PHO3* have been found down-regulated when cells were shifted from 17 °C or 21 °C to 37 °C (GEO Samples GSM883 and GSM930) [22]. This fact is compatible with the notion that Pkh-depleted cells suffer a response similar to that triggered by heat shock. Low levels of mRNA required for the iron uptake have also been found by exposure of yeast cells to arsenite, an agent that affects the transcription of genes involved in oxidative stress defense and redox maintenance [45]. However, we cannot discard that the down-regulation of genes involved in iron uptake could be related to the recently identified connection between high levels of iron and cell death by ferroptosis, in a process that requires the Pkh1-Ypk1 signaling cascade [46].

Cells containing reduced quantities of Pkh were additionally demonstrated hypersensitive to several environmental stresses such as oxidative agents. Overexpression of the transcription factor Yap1 partially rescued the lethality displayed by Pkh-deficient cells, suggesting that these cells suffer oxidative stress, and the observed transcriptional response cannot cope with it. These results are in agreement with i) the high levels of ROS found in Pkh-deficient cells [15] and ii) the hypersensitivity of Pkh-depleted cells to the oxidizing agents diamide and hydrogen peroxide. The fact that depletion of Pkh did not affect the growth in the presence of menadione is not completely surprising since cells have diverse mechanisms to protect against different oxidizing agents and none of the oxidants is representative of general oxidative stress. For example, it has been described that the single mutation of 78 genes confers sensitivity to diamide and hydrogen peroxide but not to menadione [47]. Cells containing low levels of Pkh are also hypersensitive to other stresses tested such as lithium and sodium ions and to UPR-inducers.

Interestingly, expression of *INO1* increased about 95-fold in SDP8 cells incubated with doxycycline for 24 h. Semiquantitative RT-PCR analysis confirmed a strong expression induction (Additional file 3: Figure S1). Ino1 is required for the synthesis of phosphoinositides and inositol phosphates, playing central roles in membrane traffic and signal transduction pathways. Although mammalian GST-PDK1 binds *in vitro* to several phosphoinositides, we have found that neither yeast GST-Pkh1 or GST-Pkh2 bind to any phosphoinositide under the same conditions (unpublished results). The impaired endocytosis observed in Pkh-deficient cells [48] and the role of phosphoinositides in membrane trafficking [49] could explain the high expression level of *INO1* found in Pkh-depleted cells.

Transcriptional changes observed by incubation of SDP8 cells with doxycycline for 24 h could also be due to indirect effects caused by the decreased levels of Pkh. A plausible explanation for the transcription pattern observed in Pkh-depleted cells is based in the fact that yeast PKA inhibits Msn2/Msn4 and Hsf1 transcription factors [32, 50]. Since it has been recently shown that Pkh is necessary for the activation of PKA [10, 12], cells depleted of Pkh would have lower-than-normal levels of PKA activity. Low PKA activity would lead to activation of the Msn2/Msn4 and Hsf1 transcription factors, increasing thus, the expression of the genes required for stress defense.

Pkh proteins were also found necessary for the complete transcriptional response triggered by heat stress. We have identified a set of genes whose up-regulation after stress depends on the presence of normal levels of Pkh. Expression levels of a sub-set of these genes were already increased in Pkh-depleted cells, and this could avoid further elevation of their expression in Pkh-depleted cells under heat shock conditions. However, we have found a relevant set of genes with impaired up-regulation in Pkh-deficient cells that could be considered as directly or indirectly affected by the lack of Pkh after heat stress (their expression is not increased in Pkh-depleted cells). We conclude thus that Pkh proteins influence, directly or indirectly, the transcriptional response elicited by heat stress. This is not completely unexpected, since Pkh can be associated with heat shock adaptation in different ways. First, the kinase activity of Pkh was reported to increase under heat shock stress [51]. Second, Pil1 and Lsp1, two components of eisosomes important for heat stress tolerance [52], are phosphorylated by Pkh under heat stress [51]. Third, the PH domain-containing proteins Slm1 and Slm2, regulators of actin cytoskeleton organization in response to stress, are also substrates of Pkh activity under heat shock conditions [53]. Fourth, the phosphorylation status of the Orm1 and 2 proteins, substrates for the Pkh-Ypk cascade, regulate the sphingolipid biosynthesis in response to heat stress [54]. In addition, Pkh-depleted cells are hypersensitive to incubation at 37 °C [15]. All these data indicate that the use of the temperature sensitive allele of Pkh might not be appropriate for some studies since Pkh proteins seems to be required for the proper heat stress response. Our work shows that the depletion of Pkh affects the transcription of numerous genes involved in a broad spectrum of cellular activities. Although the methodology of depletion of Pkh employed here cannot clearly distinguish direct from indirect effects, the study reveals the overall effects of Pkh depletion which is relevant for understanding the broad functions of these kinases. Since *Candida*, *Aspergilus* and other fungal infectious organisms have conserved the Pkh signaling pathway, inhibition of Pkh will also produce pleiotropic effects. In *Candida* sp there is only one gene, *CaPKH2*, representing *S. cerevisiae PKH1/2*. We have determined, in our previous work, that there are differences at the ATP-binding site and at the PIF-pocket regulatory sites that can enable the development of drugs that inhibit CaPkh2 without affecting the human ortholog PDK1 [15]. Together, our results point to fungal Pkh orthologs for selective anti-fungal drug discovery.

## Conclusion

Our studies show that depletion of Pkh affects the transcription of more than four hundred genes, increasing the mRNA levels of genes involved in the response to heat shock and oxidative stress. A relevant fraction of these up-regulated genes are known to be transcribed by the Msn2, Msn4 and Hsf1 transcription factors, suggesting that the absence of Pkh mimics a cellular response to a diversity of stresses. Our system allows, for the first time, the characterization of the transcriptomic changes caused by heat stress in Pkh-deficient cells, since the use of a thermosensitive allele did not allow such study. Thus, we demonstrate that Pkh is required for the proper transcriptional response after heat stress, although the down-regulation of genes coding for ribosomal proteins is independent of Pkh.

## Methods

### Yeast strains and culture conditions

Yeast strains used in this study (Table 1) were derivatives of the wild type CML476 [55] and have been already characterized in a previous study [15]. Yeast cells were grown at 28 °C in solid or liquid YPD medium (10 g/l yeast extract, 20 g/l peptone and 20 g/l glucose). Sensitivity of yeast cells to several agents (oxidizing, salts and UPR-inducers) in solid media was evaluated by drop test growth on YPD plates as previously described [56]. Saturated liquid cultures grown in YPD medium were initially diluted until OD_660_ 0.05, and serial dilution were prepared as specified in each case. Three μl of each dilution were spotted onto the plates containing the indicated concentrations of the agents plus doxycycline and growth was registered after incubation for the indicated time at 28 °C.

### Plasmids used

The PHO84-*lacZ* reporter is the YEp357 plasmid containing the region comprising from −603 to +19 from the *PHO84* gene fused to LacZ [57]. The STRE(7x)-*lacZ* reporter system was prepared as previously described [58]. The pMCZ-Y plasmid contains the UPRE fused to *lacZ* gene [59]. To express Yap1 the promoter and coding regions of the *YAP1* gene and GFP were cloned into the pRS316 plasmid as follows: yeast cells were co-transformed with the next three overlapping DNA fragments: A) a 2.6 kbp PCR-amplified genomic fragment, using the primers YAP1-GFP_fw2 and YAP1-GFP_rv1 (Table 4) as a forward and reverse respectively, containing the promoter and coding region of *YAP1*. B) A 0.9 kbp PCR-amplified product using the plasmid pFA6a-GFP(S65T)-HIS3MX6 [60] as a template and the primers GFP_UP and GFP_rv1 (Table 4). C) And the EcoRI/XhoI digested pRS316 plasmid [61]. Transformed cells were selected in medium without uracil and their plasmid purified and verified by restriction analysis. The functionality of the final construct was verified by its ability to complement a *yap1* deletion mutant and by visualization of the translocation to the nucleus of the Yap1-GFP fusion protein under oxidative stress.

**Table 4:** Oligonucleotides used in this study

Name	DNA sequence (5′ to 3′)
YAP1-GFP_fw2	ccgctctagaactagtggatcccccgggctgcaggaattccgaagtggagcagtttacag
YAP1-GFP_rv1	gaattgggacaactccagtgaaaagttcttctcctttactgttcatatgcttattcaaagc
GFP_UP	agtaaaggagaagaacttttcactg
GFP_rv1	ctaaagggaacaaaagctgggtaccgggccccccctcgagttaccctgttatccctagcg
FW_HXT2_RT	tgagacagaacaggagcc
RV_HXT2_RT	cacatcagacaagctagcg
RT_HXT7_UP	tgcttccgtgggtgtcacc
RT_HXT7_DOWN	tggcttgtcatcgtgagtc
RT_MDH2_UP	ggtggtatcgggcagtcg
RT_MDH2_ DOWN	cagggacgtcaggcatgg
HXK1_5_RTPCR	tcaagaccactctgccagaa
HXK1_3_RTPCR	ttggatctttgcttgcgtca
RT-GAC1_FW	actcatgctacgcctgatgg
RT-GAC1_RV	tcgaatcatgagggatggcg
GSY1_3_RTPCR	attccaaccccattctcttcc
GSY1_5_RTPCR	tgattgaaacagaccaagcga
HXK1_5_RTPCR	tcaagaccactctgccagaa
ARN1 For	tgagacagaacaggagcc
ARN1 Rev	cacatcagacaagctagcg
PHO84_F_RTPCR	tgctagagacggtaagccgccaa
PHO84_R_RTPCR	atgggctggaagattcaatg
RT-PHO11_12_FW	tcctgcttgggacgatgatg
RT-PHO11_12_RV	tcatagcctggtcccgtttg
RT-PHO5_FW	tcccttaggcaaactagccg
RT-PHO5_RV	gagccgttgaattgacgagtg
HSP12For	atgtctgacgcaggtagaaaag
HSP12 Rev	ccttcagcgttatccttgcc
HSP26 For	ggcggcttaagaggctac
HSP26 Rev	gacaccaggaaccacgac
RT-INO1_FW	ctccactttagtggcctcgg
RT-INO1_RV	ttcgccttcaagcgttgttg
RT_TUB2_For	caggtcagtgtggtaacc
RT_TUB2_Rev	ttggcccacacgttgccc

### Cultures treatments for RNA preparation

Cultures of the wild-type CML476 and its derivative SDP8 strains grown for 8 h in the presence of doxycycline (100 μg/ml) were collected at OD_660_ ~ 0.6. For the 24-h time point the appropriate quantity of cells were collected after 8 h of incubation with doxycycline, resuspended in 50 ml of YPD at an OD_660_ of 0.01 and grown for an additional 16 h in the presence of the same quantity of fresh antibiotic. For the heat-stressed cells, wild-type and SDP8 cells treated with doxycycline for 24 h as above were incubated either at 40 °C or 28 °C for 40 min. In all cases, cell samples were collected by sedimentation (5 min at 1500 g), washed with cold water, and the dried cell pellet was kept at −80 °C until RNA purification.

### Purification of RNA, RT-PCR and microarray analysis

Extraction of total RNA and transcription analysis by DNA microarrays were performed essentially as previously described [62] using 8 μg of total RNA and an indirect labeling kit (CyScribe Post-Labeling kit, GE-Amersham Biosciences) for the cDNA synthesis, in conjunction with Cy3-dUTP and Cy5-dUTP fluorescent nucleotides. The yeast genomic microchips were constructed in our laboratory and contain at least two copies for each of the 6014 different PCR-amplified ORFs from *S. cerevisiae* [GEO Platform GPL10039] [63, 64]. Pre-hybridization, hybridization, and washes were carried out as recommended by The Institute for Genomic Research [65] with the minor modifications described in [62]. The slides were scanned with a ScanArray 4000 apparatus (Packard BioChips Technologies), and the output was analyzed using GenePix Pro 6.0 software. Spots with either a diameter smaller than 120 μm or fluorescence intensities for Cy3 and Cy5 lower than 150 units were not considered for further analysis. A technical replica was performed for each experiment, where dyes were swapped to avoid dye-specific bias.

In a first set of experiments, we compared the expression profile of SDP8 cells with that of wild-type CML476 cells; both cell types were exposed to doxycycline for a total time of 8 or 24 h in YPD media. In the second series of experiments, we compared the transcriptomic profiles SDP8 and wild-type CML476 cells treated with doxycycline for 24 h and heat-shocked for 40 minutes at 40 °C with those of the equally-treated cells but in the absence of the stress. Data from technical replicas were combined and the mean was calculated. A given gene was considered to be induced or repressed when the expression ratio was higher than 2.0 or lower than 0.50, respectively. According to the expression of the genes after heat shock in the wild-type CML476 and SDP8 strains, different levels of dependence on Pkh were defined, as previously described [23]. Thus, genes showing an SDP8/wild-type ratio of 0.67 > X > 0.50 were considered “weakly dependent” (WD), those with a ratio of 0.50 > X > 0.25 were ranked as “strongly dependent” (SD) and those with a ratio ≤0.25 were defined as “totally dependent” (TD). Similarly, genes induced more than 2.5-fold in wild-type cells and considered not induced (i.e., the ratio of stress/no stress <1.3) in SDP8 cells were also considered as TD.

Expression changes for several genes were confirmed by RT–PCR analyses using the Ready-To-Go RT–PCR Beads kit (GE Healthcare) with 50 ng of total RNA in 30 cycles. Gene-specific pairs of oligonucleotides (Table 4) were used to determine the levels of the corresponding mRNAs.

### Beta-galactosidase assays

To evaluate the promoter activity of the *PHO84,* as well as the STRE- and the UPRE-driven transcriptional response in the presence and absence of doxycycline, wild-type and SDP8 cells incubated for 8 or 24 h in the presence of 100 μg/ml were harvested, β-Galactosidase activity measured as described previously [57] and results expressed as Miller Units [66]. Activation of the unfolded protein response pathway was assessed in the same way by using the pMCZ-Y plasmid contains the UPRE fused to *lacZ* gene.

### Informatics tools

The ProtParam algorithm at the Expasy server was used to estimate the protein half-life [67]. The pre-processing tool of the Babelomics platform (http://v4.babelomics.org/), was used to pre-process the microarray data, obtaining only one expression value for each gene [68]. The MIPS Functional Catalogue Database (MIPS FunCatDB) [69], available http://mips.helmholtz-muenchen.de/funcatDB/, was used for the functional distribution of gene lists. The Publication Enrichment tool available at the YeastMine website [70] was used to identified significant overlaps between customized set of genes and genes from published expression patterns. The YEASTRACT (Yeast Search for Transcriptional Regulators And Consensus Tracking; http://www.yeastract.com/) tools were used for grouping genes based on their regulatory associations with documented transcription factors [71]. We searched for transcription factors as activators and filtrated for DNA binding plus expression evidence.

### Availability of supporting data

The data set supporting the results of this article is available in the Gene Expression Omnibus repository, [GSE32623, http://www.ncbi.nlm.nih.gov/geo/query/acc.cgi?acc=GSE32623].

## Additional files

Additional file 1: Table S1. Genes up-regulated by depletion of Pkh. Data are the fold increase of the expression values for each gene in SDP8 cells incubated in the presence of doxycycline for 8 and 24 h compared to the values obtained in wild-type CML476 cells under the same treatment. (PDF 300 kb) Additional file 2: Table S2. Genes down-regulated by depletion of Pkh. Data are the fold decrease of the expression values for each gene in SDP8 cells incubated in the presence of doxycycline for 8 and 24 h compared to the values obtained in wild-type CML476 cells under the same treatment. (PDF 257 kb) Additional file 3: Figure S1. Semiquantitative analysis by RT-PCR of mRNA levels of a representative set of genes identified as altered by depletion of Pkh using microarrays analysis. RT-PCR was performed on total RNA isolated from the CML476 (WT) and SDP8 strains grown in YPD in the presence of doxycycline (100 μg/ml) for 8 and 24 h using specific sets of primers (Table 4). The numbers in brackets indicate the fold change (SDP8/WT) detected by microarrays for each gene at 8 and 24 h of incubation with doxycycline. RT-PCR of *TUB2* is shown as a control. (PPTX 466 kb) Additional file 4: Table S3. Expression changes of the ESR genes and their paralogs by depletion of Pkh. Data are the fold change of the expression values for ESR and non-ESR paralog genes in SDP8 cells incubated in the presence of doxycycline for 8 and 24 h compared to the values obtained in wild-type CML476 cells under the same treatment. (PDF 292 kb) Additional file 5: Figure S2. Transcription factors mainly affected by depletion of Pkh. **A**: Transcription factors mainly involved in controlling the sets of genes found up- and down-regulated (left and right panel, respectively) when SDP8 were grown in the presence of doxycycline for 8 h. Transcription factors with more than 6 or 5 target genes (for up- and down-regulated, respectively) were selected. Only those with a ration of % of predicted/% of expected targets genes ≥ 3 are represented. **B**: As in **A**, but for the sets of genes found up- and down-regulated when cells were incubated with doxycycline for 24. Empty bars represent the % of up-regulated genes; filled bars denote the % of down-regulated genes and striped bars indicate the % of expected genes regulated by each transcription factor. Numbers on the top of empty bars denote the ratios % of up-regulated genes/% of expected number of genes regulated by each transcription factor. Numbers on the top of filled bars indicate the ratio of % of down-regulated genes/% of expected number of genes regulated by each transcription factor. (PPTX 91 kb) Additional file 6: Figure S3. The transcriptional changes caused by heat shock are attenuated in Pkh-depleted cells. **A**: Graphical representation of the expression values (in log_2_) for the top 100 most up-regulated genes, after heat shock, in doxycycline-treated wild-type (WT) cells (○) and the corresponding value for the same genes in the SDP8 strain under the same condition (■). **B**: Similar representation for the top 100 most down-regulated genes after heat shock in doxycycline-treated wild-type (WT) cells (○) and the corresponding value for the same genes in the SDP8 strain under the same condition (■). **C**: Graphical representation of the expression values (in log_2_) for the top 100 most up-regulated genes by heat shock in doxycycline-treated SDP8 cells (■) and the corresponding value for the same genes in the wild-type (WT) strain under the same condition (○). **D**: Graphical representation of the expression values (in log_2_) for the top 100 most down-regulated genes, after heat shock, in doxycycline-treated SDP8 cells (■) and the corresponding value for the same genes in the wild-type (WT) strain under the same condition (○). (PPTX 87 kb) Additional file 7: Figure S4. Analysis of the genes up-regulated by heat shock in a Pkh-dependent manner. **A**: Cluster analysis of the expression profiles of the sub-set of 52 genes that whose expression was up-regulated upon heat stress in a Pkh-dependent manner that were found already up-regulated in SDP8 cells treated with doxycycline for 24 h (“Depletion of Pkh”). Data was hierarchically clustered (complete linkage clustering, uncentered correlation) by means of the Gene Cluster (v. 2.11) software [57] and visualized with Java TreeView (v 3.0) [58]. **B**: Cluster analysis, performed as in A, but with the sub-set of 91 genes whose expression was also up-regulated upon heat stress in a Pkh-dependent manner but that were not found up-regulated in SDP8 cells treated with doxycycline for 24 h (“Depletion of Pkh”). (PPTX 79 kb) Additional file 8: Table S4. Genes whose up-regulation by heat stress was found to be dependent on the presence of Pkh. The values correspond to the -fold change of the expression levels triggered by heat shock in wild-type (WT) and SDP8 cells grown in the presence of doxycycline (100 μg/ml) for 24 h. Dependences on Pkh are defined as TD: totally dependent, SD: Strongly dependent, WD: weakly dependent. (PDF 74 kb) Additional file 9: Table S5. Genes whose down-regulation by heat stress was found to be dependent on the presence of Pkh. The values correspond to the -fold change of the expression levels triggered by heat shock in wild-type (WT) and SDP8 cells grown in the presence of doxycycline (100 μg/ml) for 24 h. Dependences on Pkh are defined as TD: totally dependent, SD: Strongly dependent, WD: weakly dependent. (PDF 32 kb) Additional file 10: Table S6. Major functional categories of genes up- and down-regulated by heat stress. The set of genes in each category is classified as affected (dependent) or unaffected (independent) by the absence of Pkh. (PDF 21 kb)

## Abbreviations

CWI: cell wall integrity
ESR: environmental stress response
ESRE: environmental stress response element
PDK1: 3-phosphoinositide-dependent kinase
PH: pleckstrin homology
ROS: reactive oxygen species
STRE: stress response element
UPR: unfolded protein response
UPRE: unfolded protein response element

## References

[1] Zhang X, Lester RL, Dickson RC (2004). Pil1p and Lsp1p negatively regulate the 3-phosphoinositide-dependent protein kinase-like kinase Pkh1p and downstream signaling pathways Pkc1p and Ypk1p. J Biol Chem.

[2] Walther TC, Aguilar PS, Frohlich F, Chu F, Moreira K, Burlingame AL (2007). Pkh-kinases control eisosome assembly and organization. EMBO J.

[3] Bayascas JR (2010). PDK1: the major transducer of PI 3-kinase actions. Curr TopMicrobiol Immunol.

[4] Arencibia JM, Pastor-Flores D, Bauer AF, Schulze JO, Biondi RM (1834). AGC protein kinases: From structural mechanism of regulation to allosteric drug development for the treatment of human diseases. Biochim Biophys Acta.

[5] Pearce LR, Komander D, Alessi DR (2010). The nuts and bolts of AGC protein kinases. Nat Rev Mol Cell Biol.

[6] Garcia AV, Al-Yousif M, Hirt H (2012). Role of AGC kinases in plant growth and stress responses. Cell Mol Life Sci.

[7] Rademacher EH, Offringa R (2012). Evolutionary adaptations of plant AGC kinases: from light signaling to cell polarity regulation. Front Plant Sci.

[8] Casamayor A, Torrance PD, Kobayashi T, Thorner J, Alessi DR (1999). Functional counterparts of mammalian protein kinases PDK1 and SGK in budding yeast. CurrBiol.

[9] Inagaki M, Schmelzle T, Yamaguchi K, Irie K, Hall MN, Matsumoto K (1999). PDK1 homologs activate the Pkc1-mitogen-activated protein kinase pathway in yeast. Mol Cell Biol.

[10] Voordeckers K, Kimpe M, Haesendonckx S, Louwet W, Versele M, Thevelein JM (2011). Yeast 3-phosphoinositide-dependent protein kinase-1 (PDK1) orthologs Pkh1-3 differentially regulate phosphorylation of protein kinase A (PKA) and the protein kinase B (PKB)/S6K ortholog Sch9. J Biol Chem.

[11] Roelants FM, Torrance PD, Thorner J (2004). Differential roles of PDK1- and PDK2-phosphorylation sites in the yeast AGC kinases Ypk1, Pkc1 and Sch9. Microbiology.

[12] Haesendonckx S, Tudisca V, Voordeckers K, Moreno S, Thevelein JM, Portela P (2012). The activation loop of PKA catalytic isoforms is differentially phosphorylated by Pkh protein kinases in *Saccharomyces cerevisiae*. Biochem J.

[13] Huang X, Liu J, Dickson RC (2012). Down-regulating sphingolipid synthesis increases yeast lifespan. PLoS Genet.

[14] Vilaça R, Silva E, Nadais A, Teixeira V, Matmati N, Gaifem J (2014). Sphingolipid signalling mediates mitochondrial dysfunctions and reduced chronological lifespan in the yeast model of Niemann-Pick type C1. Mol Microbiol.

[15] Pastor-Flores D, Schulze JO, Bahí A, Giacometti R, Ferrer-Dalmau J, Passeron S (2013). The PIF-pocket as a target for *C. albicans* Pkh selective inhibitors. ACS Chem Biol.

[16] Kemmeren P, Sameith K, van de Pasch LAL, Benschop JJ, Lenstra TL, Margaritis T (2014). Large-scale genetic perturbations reveal regulatory networks and an abundance of gene-specific repressors. Cell.

[17] Van Wageningen S, Kemmeren P, Lijnzaad P, Margaritis T, Benschop JJ, de Castro IJ (2010). Functional overlap and regulatory links shape genetic interactions between signaling pathways. Cell.

[18] Wishart JA, Hayes A, Wardleworth L, Zhang NS, Oliver SG (2005). Doxycycline, the drug used to control the tet-regulatable promoter system, has no effect on global gene expression in *Saccharomyces cerevisiae*. Yeast.

[19] Williams-Hart T, Wu X, Tatchell K (2002). Protein phosphatase type 1 regulates ion homeostasis in *Saccharomyces cerevisiae*. Genetics.

[20] Wilson WA, Roach PJ, Montero M, Baroja-Fernández E, Muñoz FJ, Eydallin G (2010). Regulation of glycogen metabolism in yeast and bacteria. FEMS Microbiol Rev.

[21] François J, Parrou JL (2001). Reserve carbohydrates metabolism in the yeast *Saccharomyces cerevisiae*. FEMS Microbiol Rev.

[22] Gasch AP, Spellman PT, Kao CM, Carmel-Harel O, Eisen MB, Storz G (2000). Genomic expression programs in the response of yeast cells to environmental changes. Mol Biol Cell.

[23] Casamayor A, Serrano R, Platara M, Casado C, Ruiz A, Ariño J (2012). The role of the Snf1 kinase in the adaptive response of *Saccharomyces cerevisiae* to alkaline pH stress. Biochem J.

[24] Guan Q, Haroon S, Bravo DG, Will JL, Gasch AP (2012). Cellular memory of acquired stress resistance in *Saccharomyces cerevisiae*. Genetics.

[25] Du Y, Cheng W, Li WF (2012). Expression profiling reveals an unexpected growth-stimulating effect of surplus iron on the yeast *Saccharomyces cerevisiae*. Mol Cells.

[26] Shakoury-Elizeh M, Tiedeman J, Rashford J, Ferea T, Demeter J, Garcia E (2004). Transcriptional remodeling in response to iron deprivation in *Saccharomyces cerevisiae*. Mol Biol Cell.

[27] Wu WS, Li WH (2008). Identifying gene regulatory modules of heat shock response in yeast. BMCGenomics.

[28] Teng S-C, Epstein C, Tsai Y-L, Cheng H-W, Chen H-L, Lin J-J (2002). Induction of global stress response in *Saccharomyces cerevisiae* cells lacking telomerase. Biochem Biophys Res Commun.

[29] Liu X, Zhang X, Wang C, Liu L, Lei M, Bao X (2007). Genetic and comparative transcriptome analysis of bromodomain factor 1 in the salt stress response of *Saccharomyces cerevisiae*. Curr Microbiol.

[30] Gasch AP, Hohmann S, Mager WH (2002). The environmental stress response: a common yeast response to diverse environmental stresses. Yeast Stress Responses.

[31] Parrou JL, Teste MA, Francois J (1997). Effects of various types of stress on the metabolism of reserve carbohydrates in *Saccharomyces cerevisiae*: genetic evidence for a stress-induced recycling of glycogen and trehalose. Microbiology.

[32] Morano KA, Grant CM, Moye-Rowley WS (2012). The response to heat shock and oxidative stress in *Saccharomyces cerevisiae*. Genetics.

[33] Korennykh A, Walter P (2012). Structural basis of the unfolded protein response. Annu Rev Cell Dev Biol.

[34] Kimata Y, Ishiwata-Kimata Y, Yamada S, Kohno K (2006). Yeast unfolded protein response pathway regulates expression of genes for anti-oxidative stress and for cell surface proteins. Genes Cells.

[35] Dickson RC (2008). Thematic review series: sphingolipids. New insights into sphingolipid metabolism and function in budding yeast. J Lipid Res.

[36] Luo Z, van Vuuren HJ (2009). Functional analyses of PAU genes in *Saccharomyces cerevisiae*. Microbiology.

[37] Verghese J, Abrams J, Wang Y, Morano KA (2012). Biology of the heat shock response and protein chaperones: budding yeast (*Saccharomyces cerevisiae*) as a model system. Microbiol Mol Biol Rev.

[38] Casado C, González A, Platara M, Ruiz A, Ariño J (2011). The role of the protein kinase A pathway in the response to alkaline pH stress in yeast. Biochem J.

[39] Gasch AP, Huang M, Metzner S, Botstein D, Elledge SJ, Brown PO (2001). Genomic expression responses to DNA-damaging agents and the regulatory role of the yeast ATR homolog Mec1p. Mol Biol Cell.

[40] Tu BP, Weissman JS (2004). Oxidative protein folding in eukaryotes: mechanisms and consequences. J Cell Biol.

[41] Perrone GG, Tan SX, Dawes IW (2008). Reactive oxygen species and yeast apoptosis. Biochim Biophys Acta - Mol Cell Res.

[42] Grüning N-M, Rinnerthaler M, Bluemlein K, Mülleder M, Wamelink MMC, Lehrach H (2011). Pyruvate kinase triggers a metabolic feedback loop that controls redox metabolism in respiring cells. Cell Metab.

[43] Ferrer-Dalmau J, Randez-Gil F, Marquina M, Prieto JA, Casamayor A (2015). Protein kinase Snf1 is involved in the proper regulation of the unfolded protein response in *Saccharomyces cerevisiae*. Biochem J.

[44] Düvel K, Santhanam A, Garrett S, Schneper L, Broach JR (2003). Multiple roles of Tap42 in mediating rapamycin-induced transcriptional changes in yeast. Mol Cell.

[45] Thorsen M, Lagniel G, Kristiansson E, Junot C, Nerman O, Labarre J (2007). Quantitative transcriptome, proteome, and sulfur metabolite profiling of the *Saccharomyces cerevisiae* response to arsenite. Physiol Genomics.

[46] Dixon SJ, Stockwell BR (2014). The role of iron and reactive oxygen species in cell death. Nat Chem Biol.

[47] Thorpe GW, Fong CS, Alic N, Higgins VJ, Dawes IW (2004). Cells have distinct mechanisms to maintain protection against different reactive oxygen species: oxidative-stress-response genes. Proc Natl Acad Sci U S A.

[48] Friant S, Lombardi R, Schmelzle T, Hall MN, Riezman H (2001). Sphingoid base signaling via Pkh kinases is required for endocytosis in yeast. EMBO J.

[49] De Camilli P, Emr SD, McPherson PS, Novick P (1996). Phosphoinositides as regulators in membrane traffic. Science.

[50] Lee P, Cho BR, Joo HS, Hahn JS (2008). Yeast Yak1 kinase, a bridge between PKA and stress-responsive transcription factors, Hsf1 and Msn2/Msn4. Mol Microbiol.

[51] Luo G, Gruhler A, Liu Y, Jensen ON, Dickson RC (2008). The sphingolipid long-chain base-Pkh1/2-Ypk1/2 signaling pathway regulates eisosome assembly and turnover. J Biol Chem.

[52] Dickson RC, Sumanasekera C, Lester RL (2006). Functions and metabolism of sphingolipids in *Saccharomyces cerevisiae*. Prog Lipid Res.

[53] Daquinag A, Fadri M, Jung SY, Qin J, Kunz J (2007). The yeast PH domain proteins Slm1 and Slm2 are targets of sphingolipid signaling during the response to heat stress. Mol Cell Biol.

[54] Sun Y, Miao Y, Yamane Y, Zhang C, Shokat KM, Takematsu H (2012). Orm protein phosphoregulation mediates transient sphingolipid biosynthesis response to heat stress via the Pkh-Ypk and Cdc55-PP2A pathways. Mol Biol Cell.

[55] Yen K, Gitsham P, Wishart J, Oliver SG, Zhang N (2003). An improved tetO promoter replacement system for regulating the expression of yeast genes. Yeast.

[56] Ferrer-Dalmau J, González A, Platara M, Navarrete C, Martínez JL, Barreto L (2010). Ref2, a regulatory subunit of the yeast protein phosphatase 1, is a novel component of cation homoeostasis. Biochem J.

[57] Serrano R, Ruiz A, Bernal D, Chambers JR, Arino J (2002). The transcriptional response to alkaline pH in *Saccharomyces cerevisiae*: evidence for calcium-mediated signalling. Mol Microbiol.

[58] Gonzalez A, Ruiz A, Casamayor A, Arino J (2009). Normal function of the yeast TOR pathway requires the type 2C protein phosphatase Ptc1. Mol Cell Biol.

[59] Mori K, Kawahara T, Yoshida H, Yanagi H, Yura T (1996). Signalling from endoplasmic reticulum to nucleus: transcription factor with a basic-leucine zipper motif is required for the unfolded protein-response pathway. Genes Cells.

[60] Longtine MS, McKenzie A, Demarini DJ, Shah NG, Wach A, Brachat A (1998). Additional modules for versatile and economical PCR-based gene deletion and modification in *Saccharomyces cerevisiae*. Yeast.

[61] Sikorski RS, Hieter P (1989). A system of shuttle vectors and yeast host strains designed for efficient manipulation of DNA in *Saccharomyces cerevisiae*. Genetics.

[62] Gonzalez A, Ruiz A, Serrano R, Arino J, Casamayor A (2006). Transcriptional profiling of the protein phosphatase 2C family in yeast provides insights into the unique functional roles of Ptc1. J Biol Chem.

[63] Viladevall L, Serrano R, Ruiz A, Domenech G, Giraldo J, Barcelo A (2004). Characterization of the calcium-mediated response to alkaline stress in *Saccharomyces cerevisiae*. J Biol Chem.

[64] Alberola TM, Garcia-Martinez J, Antunez O, Viladevall L, Barcelo A, Arino J (2004). A new set of DNA macrochips for the yeast *Saccharomyces cerevisiae*: features and uses. Int Microbiol.

[65] Hegde P, Qi R, Abernathy K, Gay C, Dharap S, Gaspard R (2000). A concise guide to cDNA microarray analysis. Biotechniques.

[66] Miller JH (1972). Experiments in Molecular Genetics.

[67] Gasteiger E, Hoogland C, Gattiker A, Duvaud S, Wilkins MR, Appel RD et al. Protein Identification and Analysis Tools on the ExPASy Server. In Walker JM, editor. The Proteomics Protocols Handbook. Humana Press; 2005. p571–607.

[68] Herrero J, Al Shahrour F, Diaz-Uriarte R, Mateos A, Vaquerizas JM, Santoyo J (2003). GEPAS: A web-based resource for microarray gene expression data analysis. Nucleic Acids Res.

[69] Ruepp A, Zollner A, Maier D, Albermann K, Hani J, Mokrejs M (2004). The FunCat, a functional annotation scheme for systematic classification of proteins from whole genomes. Nucleic Acids Res.

[70] Balakrishnan R, Park J, Karra K, Hitz BC, Binkley G, Hong EL (2012). YeastMine--an integrated data warehouse for *Saccharomyces cerevisiae* data as a multipurpose tool-kit. Database.

[71] Abdulrehman D, Monteiro PT, Teixeira MC, Mira NP, Lourenço AB, dos Santos SC (2011). YEASTRACT: providing a programmatic access to curated transcriptional regulatory associations in *Saccharomyces cerevisiae* through a web services interface. Nucleic Acids Res.

[72] Eisen MB, Spellman PT, Brown PO, Botstein D (1998). Cluster analysis and display of genome-wide expression patterns. Proc Natl Acad Sci U S A.

[73] Saldanha AJ (2004). Java Treeview--extensible visualization of microarray data. Bioinformatics.

[74] Cherry JM, Hong EL, Amundsen C, Balakrishnan R, Binkley G, Chan ET (2012). Saccharomyces Genome Database: the genomics resource of budding yeast. Nucleic Acids Res.

[75] Schröder M, Clark R, Kaufman RJ (2003). IRE1- and HAC1-independent transcriptional regulation in the unfolded protein response of yeast. Mol Microbiol.

