# Supplementary material for: Depletion of yeast PDK1 orthologs triggers a stress-like transcriptional response
Source: BMC Genomics. 2015 Sep 21;16(1):719. doi: 10.1186/s12864-015-1903-8 (PMC4578605; doi:10.1186/s12864-015-1903-8)
Supplement: Additional file 1: Table S1. — Genes up-regulated by depletion of Pkh. Data are the fold increase of the expression values for each gene in SDP8 cells incubated in the presence of doxycycline for 8 and 24 h compared to the values obtained in wild-type CML476 cells under the same treatment. (PDF 300 kb) [file 12864_2015_1903_MOESM1_ESM.pdf]

Supporting Table S1: Genes up-regulated by depletion of Pkh.

INDUCED GENES				
ORF	GENE	-fold increase		INFO
		at 8h	at 24h	
YAL054C	*ACS1*		**12.32**	1,2,4
YAL061W	*BDH2*	**2.66**	**4.19**	1,2,6,8
YAR028W		**1.05**	**2.56**	
YAR035W	*YAT1*		**2.92**	2
YBL015W	*ACH1*	**1.40**	**2.60**	2
YBL042C	*FUI1*		**3.42**	
YBL064C	*PRX1*	**1.85**	**2.86**	3,6,8
YBL075C	*SSA3*		**8.28**	3,5,8
YBL078C	*ATG8*		**4.98**	7,8
YBR005W	*RCR1*	**2.05**	**1.87**	
YBR006W	*UGA2*		**2.07**	2,3,6,8
YBR008C	*FLR1*	**0.94**	**2.58**	
YBR050C	*REG2*		**4.78**	2
YBR056W		**1.72**	**2.30**	2,6,7,8
YBR072W	*HSP26*	**6.07**	**7.47**	3,6,8
YBR083W	*TEC1*	**2.95**		
YBR101C	*FES1*	**0.58**	**2.66**	
YBR112C	*CYC8*	**1.18**	**2.57**	2
YBR117C	*TKL2*		**9.88**	1,2,5,6
YBR126C	*TPS1*	**1.53**	**2.02**	1,2,3,6,8
YBR132C	*AGP2*	**2.43**	**1.22**	4
YBR169C	*SSE2*	**1.03**	**2.80**	3,5,6,8
YBR203W	*COS111*		**2.44**	
YBR214W	*SDS24*	**1.65**	**2.89**	6
YBR230C	*OM14*	**2.37**	**2.73**	4,6,8
YBR284W			**3.02**	1
YBR287W		**1.64**	**2.34**	8
YBR298C	*MAL31*	**3.13**		4
YCL040W	*GLK1*	**1.84**	**2.02**	1,2,4,6,7,8
YCR021C	*HSP30*	**0.71**	**5.81**	3
YCR051W		**2.01**	**2.20**	
YCR091W	*KIN82*	**2.10**		4,8
YCR102C			**5.51**	1,2
YCR107W	*AAD3*	**0.69**	**4.11**	1,2
YCRX21C			**3.68**	
YDL014W	*NOP1*		**2.77**	2
YDL085W	*NDE2*		**4.34**	1,6
YDL113C	*ATG20*	**1.71**	**2.06**	
YDL124W		**1.63**	**2.32**	2,5,6,7,8
YDL149W	*ATG9*		**2.07**	1
YDL169C	*UGX2*		**3.47**	6
YDL174C	*DLD1*	**1.97**	**4.20**	1,2
YDL204W	*RTN2*	**1.33**	**2.43**	5,6,8
YDL222C	*FMP45*		**12.70**	6,8
YDL223C	*HBT1*		**5.00**	6
YDL233W		**1.67**	**2.25**	
YDL234C	*GYP7*	**2.75**	**3.68**	7
YDR011W	*SNQ2*	**1.01**	**2.21**	2,3
YDR043C	*NRG1*	**1.58**	**3.14**	1,2,3

INDUCED GENES				
ORF	GENE	-fold increase		INFO
		at 8h	at 24h	
YDR055W	*PST1*	**1.06**	**3.01**	
YDR059C	*UBC5*	**1.26**	**2.02**	3,6,8
YDR070C	*FMP16*		**5.90**	5,6,8
YDR085C	*AFR1*	**2.05**	**1.88**	8
YDR096W	*GIS1*	**2.12**	**1.82**	4,8
YDR123C	*INO2*		**2.35**	
YDR171W	*HSP42*	**1.16**	**2.04**	3,7,8
YDR178W	*SDH4*	**1.69**	**2.27**	1,2
YDR216W	*ADR1*		**4.68**	1,2,4
YDR223W	*CRF1*		**3.05**	
YDR256C	*CTA1*	**1.01**	**2.05**	3
YDR258C	*HSP78*	**1.02**	**2.18**	3,5,8
YDR342C	*HXT7*	**4.39**	**2.30**	2,4
YDR380W	*ARO10*		**13.25**	1,2,6,8
YDR453C	*TSA2*		**2.24**	3,6,7,8
YDR533C	*HSP31*	**1.42**	**4.50**	3,5,6,8
YEL011W	*GLC3*	**5.01**	**3.81**	1,2,4,8
YEL039C	*CYC7*	**2.72**		1,8
YEL045C		**1.10**	**2.10**	
YEL049W	*PAU2*		**2.90**	3
YEL060C	*PRB1*	**2.08**	**3.55**	3,8
YER020W	*GPA2*	**2.04**	**2.17**	
YER053C	*PIC2*	**1.03**	**2.64**	6,8
YER054C	*GIP2*		**3.43**	1,2
YER067W		**5.72**	**3.61**	4
YER103W	*SSA4*		**18.71**	3,5,6,8
YER143W	*DDI1*	**1.05**	**2.34**	3
YER150W	*SPI1*	**1.74**	**9.21**	3,5,8
YFL014W	*HSP12*		**52.73**	2,3,6,7,8
YFL016C	*MDJ1*	**0.69**	**2.26**	3
YFL054C		**2.37**	**1.91**	4
YFL056C	*AAD6*		**10.71**	1,2
YFL057C	*AAD16*		**10.58**	1,2
YFR015C	*GSY1*	**5.35**	**4.14**	1,2,4
YFR053C	*HXK1*	**4.84**	**3.86**	1,2,6,8
YGL006W	*PMC1*	**1.53**	**2.96**	8
YGL053W	*PRM8*	**1.46**	**2.11**	
YGL055W	*OLE1*	**1.24**	**2.66**	
YGL121C	*GPG1*		**2.34**	5,8
YGL156W	*AMS1*		**2.45**	2,8
YGL231C	*EMC4*	**2.10**		
YGR008C	*STF2*	**2.32**	**2.27**	1,3,7,8
YGR032W	*GSC2*	**2.01**	**2.10**	1,2,7
YGR043C	*NQM1*		**8.04**	1,2,5,6,8
YGR110W	*CLD1*		**3.72**	4
YGR142W	*BTN2*		**22.19**	5
YGR197C	*SNG1*	**1.19**	**2.88**	
YGR201C			**3.37**	6,8
YGR213C	*RTA1*		**4.71**	

INDUCED GENES				
ORF	GENE	-fold increase		INFO
		at 8h	at 24h	
YGR243W	*FMP43*	**2.97**	**4.02**	1,3,4,6
YGR248W	*SOL4*	**2.66**	**5.90**	1,6,8
YGR249W	*MGA1*	**2.11**		
YGR256W	*GND2*		**4.00**	1,2,6,8
YHL021C	*AIM17*	**3.14**	**3.50**	2,8
YHL027W	*RIM101*	**1.44**	**2.13**	3
YHR008C	*SOD2*	**1.17**	**2.01**	3,6
YHR033W			**2.24**	6
YHR087W	*RTC3*	**2.65**	**6.42**	6,7,8
YHR092C	*HXT4*	**6.37**		4
YHR096C	*HXT5*		**7.88**	5,6,7
YHR104W	*GRE3*	**1.23**	**2.28**	2,3,5,6,7,8
YHR138C		**2.17**	**2.86**	6,7,8
YHR139C	*SPS100*	**0.96**	**4.50**	3,6
YHR209W	*CRG1*	**1.72**	**2.35**	2
YIL017C	*VID28*	**1.55**	**2.04**	1
YIL136W	*OM45*	**2.07**		6,8
YIL144W	*TID3*	**1.18**	**4.11**	
YIL169C		**2.02**	**0.78**	
YIR016W		**2.09**	**1.70**	
YJL016W			**3.51**	5
YJL048C	*UBX6*		**4.09**	6,8
YJL066C	*MPM1*	**1.41**	**2.10**	8
YJL116C	*NCA3*	**0.80**	**2.22**	2,3,5
YJL141C	*YAK1*	**1.96**	**2.71**	3,8
YJL153C	*INO1*		**95.70**	2,5,7
YJL165C	*HAL5*	**1.89**	**3.45**	3
YJL166W	*QCR8*	**2.21**		1
YJL219W	*HXT9*		**2.74**	2
YJL221C	*FSP2*		**2.19**	1,2
YJR073C	*OPI3*	**1.94**	**3.30**	
YJR096W			**2.88**	2,8
YJR115W		**4.14**		
YKL001C	*MET14*	**1.87**	**2.68**	7
YKL062W	*MSN4*	**2.23**	**1.41**	
YKL071W		**0.95**	**7.36**	2
YKL087C	*CYT2*	**1.16**	**2.32**	
YKL109W	*HAP4*	**2.08**	**2.14**	2,4
YKL150W	*MCR1*	**1.68**	**2.62**	1,3,6,8
YKL163W	*PIR3*		**2.67**	3,5
YKL216W	*URA1*	**3.93**	**4.27**	
YKL218C	*SRY1*	**1.41**	**2.19**	
YKR024C	*DBP7*	**1.14**	**2.78**	
YKR058W	*GLG1*	**2.15**		1,4,8
YKR061W	*KTR2*	**1.22**	**2.08**	2
YKR075C		**6.89**	**4.80**	4
YKR076W	*ECM4*	**2.47**	**2.86**	6,8
YKR091W	*SRL3*	**2.40**	**1.89**	
YKR097W	*PCK1*		**8.44**	1,2

INDUCED GENES				
ORF	GENE	-fold increase		INFO
		at 8h	at 24h	
YKR098C	*UBP11*	**1.45**	**2.81**	4
YLR054C	*OSW2*		**8.44**	
YLR120C	*YPS1*	**2.17**	**2.99**	7
YLR142W	*PUT1*	**5.31**	**4.68**	8
YLR149C		**1.81**	**3.14**	4,8
YLR174W	*IDP2*		**3.77**	1,2
YLR177W		**2.19**	**1.47**	
YLR178C	*TFS1*	**2.73**	**5.41**	5,6,7,8
YLR194C		**2.53**	**5.92**	7
YLR216C	*CPR6*	**0.87**	**2.32**	3,5
YLR257W		**1.85**	**2.74**	7
YLR258W	*GSY2*	**2.26**	**1.88**	1,8
YLR267W	*BOP2*		**2.46**	4,8
YLR272C	*YCS4*	**2.33**	**6.68**	
YLR277C	*YSH1*	**1.15**	**2.81**	
YLR282C		**3.39**	**3.25**	
YLR294C		**2.27**		
YLR312C			**3.90**	6,8
YLR327C	*TMA10*	**2.77**	**2.67**	1,3,4,5,6,8
YLR331C	*JIP3*	**2.89**	**2.05**	
YLR350W	*ORM2*	**1.61**	**2.10**	3,7
YLR392C	*ART10*		**2.50**	
YLR414C		**3.25**	**6.25**	
YLR460C		**1.18**	**6.32**	1,2
YLL026W	*HSP104*	**1.04**	**3.69**	3,5,7,8
YLL039C	*UBI4*	**1.71**	**2.14**	3,8
YLL056C			**4.80**	
YML118W	*NGL3*		**3.25**	1
YML128C	*MSC1*	**2.91**	**8.54**	6,8
YMR008C	*PLB1*	**1.50**	**2.16**	
YMR011W	*HXT2*	**9.13**	**7.44**	2,4
YMR020W	*FMS1*	**1.43**	**2.42**	2
YMR030W	*RSF1*		**2.35**	1
YMR081C	*ISF1*	**4.75**	**3.49**	1,8
YMR084W			**8.43**	1,2
YMR085W			**4.79**	1,2
YMR090W			**4.18**	6,8
YMR096W	*SNZ1*	**0.63**	**3.72**	3
YMR105C	*PGM2*	**4.20**	**3.66**	1,2,6,8
YMR110C	*HFD1*	**1.83**	**2.33**	2,3,8
YMR114C		**1.82**	**2.66**	
YMR135C	*GID8*	**1.51**	**2.00**	1
YMR136W	*GAT2*	**2.31**	**10.53**	8
YMR140W	*SIP5*		**2.07**	2,3
YMR169C	*ALD3*		**15.20**	1,2,3,6,8
YMR170C	*ALD2*	**1.46**	**7.73**	1,2,3,6,8
YMR180C	*CTL1*	**0.85**	**2.02**	
YMR250W	*GAD1*	**2.03**	**4.59**	3,5,6,8
YMR280C	*CAT8*		**5.48**	1,2,4

INDUCED GENES				
ORF	GENE	-fold increase		INFO
		at 8h	at 24h	
YMR284W	*YKU70*		**2.11**	3
YMR291W		**2.27**	**1.11**	4,8
YMR304C-A		**1.44**	**2.39**	7
YMR316C-A		**2.62**	**5.04**	
YNL013C			**4.87**	
YNL014W	*HEF3*		**2.09**	
YNL015W	*PBI2*	**1.20**	**2.41**	7,8
YNL036W	*NCE103*	**0.85**	**2.83**	2,7
YNL077W	*APJ1*	**0.80**	**3.64**	3
YNL093W	*YPT53*		**6.67**	
YNL134C		**1.60**	**3.56**	1,8
YNL144C		**2.51**	**2.16**	4
YNL192W	*CHS1*	**1.81**	**2.71**	2,3
YNL195C		**1.46**	**2.51**	5,8
YNL208W		**1.60**	**2.14**	6,7,8
YNL305C		**1.65**	**2.02**	8
YNL331C	*AAD14*		**2.61**	1,2
YNR002C	*ATO2*		**6.64**	2,8
YNR059W	*MNT4*		**2.28**	2,7
YNR064C			**3.46**	2,5
YOL016C	*CMK2*	**2.81**	**4.38**	7
YOL032W	*OPI10*	**1.07**	**2.47**	7
YOL052C-A	*DDR2*		**5.39**	3
YOL084W	*PHM7*		**18.34**	5,6,8
YOL126C	*MDH2*	**3.49**	**3.66**	1,2
YOL143C	*RIB4*	**2.11**	**1.83**	
YOL165C	*AAD15*	**1.33**	**2.21**	1,2
YOR019W		**2.18**		
YOR028C	*CIN5*		**5.17**	3
YOR036W	*PEP12*	**2.08**	**2.39**	7
YOR065W	*CYT1*	**1.41**	**2.59**	1
YOR120W	*GCY1*	**2.21**	**5.03**	2,3,5
YOR121C			**5.74**	
YOR134W	*BAG7*		**5.73**	
YOR137C	*SIA1*	**2.35**		
YOR162C	*YRR1*	**1.35**	**2.65**	3
YOR178C	*GAC1*	**3.88**	**3.52**	1,2,3
YOR202W	*HIS3*	**9.34**	**10.29**	
YOR208W	*PTP2*		**2.74**	3,5,7
YOR220W	*RCN2*	**2.36**	**3.94**	6,7,8
YOR273C	*TPO4*	**3.23**	**5.80**	8
YOR289W		**2.40**	**1.95**	
YOR347C	*PYK2*	**2.06**	**1.03**	1,4
YOR374W	*ALD4*	**2.06**	**2.68**	1,2,4,8
YOR385W		**2.08**	**4.08**	
YOR391C	*HSP33*		**2.18**	3,5
YPL057C	*SUR1*	**2.95**	**2.42**	2,4
YPL061W	*ALD6*	**1.38**	**3.44**	1,2,4
YPL070W	*MUK1*	**1.11**	**2.60**	

INDUCED GENES				
ORF	GENE	-fold increase		INFO
		at 8h	at 24h	
YPL149W	*ATG5*	**1.48**	**2.20**	
YPL186C	*UIP4*	**1.61**	**2.53**	8
YPL195W	*APL5*	**1.96**	**2.22**	
YPL196W	*OXR1*	**1.51**	**2.24**	3,8
YPL223C	*GRE1*		**6.23**	3,5,6
YPL240C	*HSP82*	**0.66**	**2.07**	1,3,5
YPR001W	*CIT3*		**4.59**	1,2,4
YPR005C	*HAL1*		**2.32**	3,4
YPR030W	*CSR2*	**2.37**	**6.03**	4
YPR093C	*ASR1*	**1.29**	**2.18**	3
YPR150W			**10.86**	6,8
YPR154W	*PIN3*	**1.18**	**2.43**	

1: Energy (FunCat 02)
2: C-compound and carbohydrate metabolism (FunCat 01.05)
3: Stress response (FunCat 32.01)
4: Induced by Quinine (dos Santos et al,2009)
5: Induced by mild heat stress (Teng et al, 2002)
6: Induced by salt treatment (Liu et al, 2007)
7: Induced by tunicamycin (Kimata et al, 2006)
8: Induced ESR gene (Gasch et al, 2000)
