# Supplementary material for: Depletion of yeast PDK1 orthologs triggers a stress-like transcriptional response
Source: BMC Genomics. 2015 Sep 21;16(1):719. doi: 10.1186/s12864-015-1903-8 (PMC4578605; doi:10.1186/s12864-015-1903-8)
Supplement: Additional file 2: Table S2. — Genes down-regulated by depletion of Pkh. Data are the fold decrease of the expression values for each gene in SDP8 cells incubated in the presence of doxycycline for 8 and 24 h compared to the values obtained in wild-type CML476 cells under the same treatment. (PDF 257 kb) [file 12864_2015_1903_MOESM2_ESM.pdf]

Supporting Table S2: Genes down-regulated by depletion of Pkh.

REPRESSED GENES				
ORF	GENE	-fold decrease		INFO
		at 8h	at 24h	
YAL023C	*PMT2*	**0.84**	**0.47**	
YAR071W	*PHO11*	**0.30**	**0.31**	2,3
YBR031W	*RPL4A*	**0.77**	**0.47**	4
YBR048W	*RPS11B*	**0.92**	**0.49**	4
YBR067C	*TIP1*	**0.98**	**0.41**	3
YBR069C	*TAT1*	**0.71**	**0.49**	
YBR092C	*PHO3*	**0.49**	**0.34**	2,3
YBR093C	*PHO5*	**0.37**	**0.45**	2,3
YBR158W	*AMN1*	**0.94**	**0.49**	
YBR231C	*SWC5*	**0.44**	**1.12**	
YBR238C		**0.65**	**0.36**	4
YBR249C	*ARO4*	**0.67**	**0.44**	4
YCL030C	*HIS4*	**0.51**	**0.35**	
YCR013C		**0.82**	**0.49**	
YDL003W	*MCD1*	**0.41**		
YDL061C	*RPS29B*	**0.86**	**0.45**	4
YDL083C	*RPS16B*	**0.78**	**0.48**	4
YDL157C		**0.86**	**0.48**	
YDL191W	*RPL35A*	**0.82**	**0.49**	4
YDR023W	*SES1*	**0.71**	**0.44**	4
YDR025W	*RPS11A*	**0.81**	**0.48**	4
YDR033W	*MRH1*	**0.75**	**0.27**	3
YDR240C	*SNU56*		**0.49**	
YDR300C	*PRO1*	**0.71**	**0.44**	4
YDR324C	*UTP4*	**0.49**	**0.66**	4
YDR490C	*PKH1*	**0.05**	**0.09**	
YDR508C	*GNP1*	**0.76**	**0.47**	
YDR534C	*FIT1*	**0.75**	**0.18**	1,2
YEL021W	*URA3*	**0.10**	**0.21**	
YEL022W	*GEA2*	**0.68**	**0.50**	
YEL065W	*SIT1*	**0.38**	**0.26**	1,2
YEL071W	*DLD3*	**0.41**	**0.53**	
YER011W	*TIR1*	**0.82**	**0.34**	2,3
YER056C	*FCY2*	**0.80**	**0.46**	3
YER070W	*RNR1*	**0.74**	**0.18**	4
YER082C	*UTP7*	**0.29**		4
YER131W	*RPS26B*	**0.98**	**0.42**	4
YER145C	*FTR1*	**0.55**	**0.28**	1
YFL004W	*VTC2*	**0.78**	**0.42**	
YFR031C-A	*RPL2A*	**0.81**	**0.49**	4
YGL008C	*PMA1*	**0.68**	**0.22**	1,4
YGL012W	*ERG4*	**0.81**	**0.33**	
YGL039W		**0.71**	**0.48**	
YGL103W	*RPL28*	**0.88**	**0.49**	4
YGL123W	*RPS2*	**0.79**	**0.46**	4
YGL147C	*RPL9A*	**0.84**	**0.43**	4
YGL245W	*GUS1*	**0.83**	**0.44**	
YGR035C		**0.80**	**0.43**	
YGR061C	*ADE6*	**0.67**	**0.44**	

REPRESSED GENES				
ORF	GENE	-fold decrease		INFO
		at 8h	at 24h	
YGR079W			**0.33**	
YGR108W	*CLB1*	**0.67**	**0.41**	
YGR168C		**0.82**	**0.27**	
YGR211W	*ZPR1*	**0.45**	**1.06**	
YGR234W	*YHB1*	**0.79**	**0.30**	
YGR264C	*MES1*	**0.72**	**0.47**	4
YHL035C	*VMR1*		**0.50**	
YHL040C	*ARN1*	**0.40**	**0.18**	1,2
YHL047C	*ARN2*	**0.47**	**0.18**	1,2
YHR019C	*DED81*	**0.76**	**0.48**	4
YHR020W		**0.60**	**0.38**	4
YHR068W	*DYS1*	**0.69**	**0.40**	4
YHR143W	*DSE2*	**1.10**	**0.38**	
YHR182C-A		**0.94**	**0.48**	
YHR203C	*RPS4B*		**0.49**	4
YHR215W	*PHO12*	**0.25**	**0.19**	2,3
YIL018W	*RPL2B*	**0.87**	**0.45**	4
YIL052C	*RPL34B*	**0.88**	**0.48**	4
YJL006C	*CTK2*	**1.02**	**0.44**	
YJL012C	*VTC4*	**0.64**	**0.29**	
YJL078C	*PRY3*	**0.62**	**0.44**	
YJL079C	*PRY1*	**0.85**	**0.38**	
YJL080C	*SCP160*	**0.79**	**0.49**	4
YJL138C	*TIF2*	**0.90**	**0.49**	4
YJL178C	*ATG27*		**0.47**	
YJL190C	*RPS22A*	**0.68**	**0.41**	4
YJR029W		**0.72**	**0.48**	
YJR123W	*RPS5*	**0.85**	**0.46**	4
YJR148W	*BAT2*	**0.94**	**0.48**	
YKL008C	*LAC1*	**0.90**	**0.42**	
YKL081W	*TEF4*	**0.71**	**0.47**	3,4
YKL180W	*RPL17A*		**0.35**	4
YKR103W	*NFT1*		**0.32**	
YKR104W			**0.49**	
YLR027C	*AAT2*	**0.67**	**0.46**	
YLR029C	*RPL15A*	**0.94**	**0.48**	4
YLR048W	*RPS0B*	**0.75**	**0.50**	4
YLR056W	*ERG3*	**0.66**	**0.47**	3
YLR058C	*SHM2*	**0.72**	**0.43**	
YLR073C	*RFU1*	**0.46**	**0.77**	4
YLR134W	*PDC5*	**0.65**	**0.44**	4
YLR167W	*RPS31*	**0.90**	**0.48**	4
YLR188W	*MDL1*	**0.63**	**0.44**	
YLR214W	*FRE1*	**0.50**	**0.61**	1
YLR300W	*EXG1*	**0.65**	**0.38**	
YLR304C	*ACO1*	**0.47**	**0.28**	3
YLR332W	*MID2*	**0.50**	**0.55**	
YLR339C		**0.83**	**0.40**	4
YLR340W	*RPP0*	**0.78**	**0.46**	3,4

REPRESSED GENES				
ORF	GENE	-fold decrease		INFO
		at 8h	at 24h	
YLR348C	*DIC1*	**0.73**	**0.42**	1
YLR349W		**0.80**	**0.43**	
YLR355C	*ILV5*	**0.41**	**0.50**	
YLR406C	*RPL31B*	**0.78**	**0.48**	
YLR413W		**0.69**	**0.36**	3,4
YLR441C	*RPS1A*		**0.48**	4
YLL044W		**0.72**	**0.42**	4
YML027W	*YOX1*	**0.34**		
YML052W	*SUR7*	**0.83**	**0.46**	3
YML063W	*RPS1B*	**0.91**	**0.46**	4
YML080W	*DUS1*	**0.46**	**0.66**	4
YML123C	*PHO84*	**0.34**	**0.11**	1
YMR006C	*PLB2*	**0.81**	**0.43**	2
YMR058W	*FET3*	**0.54**	**0.20**	1,3
YMR116C	*ASC1*	**0.78**	**0.49**	4
YMR120C	*ADE17*	**0.84**	**0.47**	
YMR189W	*GCV2*	**0.70**	**0.39**	
YMR205C	*PFK2*	**0.73**	**0.48**	2
YMR272C	*SCS7*	**0.91**	**0.48**	
YMR303C	*ADH2*	**0.81**	**0.45**	
YMR317W			**0.25**	
YMR319C	*FET4*	**1.07**	**0.40**	1,3
YNL066W	*SUN4*	**0.95**	**0.49**	4
YNL067W	*RPL9B*	**0.83**	**0.46**	4
YNL069C	*RPL16B*	**0.83**	**0.48**	4
YNL145W	*MFA2*		**0.33**	
YNL164C	*IBD2*	**0.49**	**1.02**	
YNL209W	*SSB2*	**0.65**	**0.49**	3,4
YNL231C	*PDR16*	**0.52**	**0.45**	
YNL289W	*PCL1*	**0.36**	**1.06**	
YNR056C	*BIO5*		**0.34**	
YNR067C	*DSE4*	**0.65**	**0.42**	
YOL058W	*ARG1*	**0.47**	**0.39**	
YOL086C	*ADH1*	**0.82**	**0.48**	
YOR009W	*TIR4*		**0.29**	2
YOR010C	*TIR2*		**0.50**	2
YOR011W	*AUS1*		**0.38**	1
YOR063W	*RPL3*	**0.80**	**0.47**	4
YOR096W	*RPS7A*	**0.80**	**0.48**	4
YOR135C	*IRC14*	**0.65**	**0.29**	
YOR136W	*IDH2*	**0.58**	**0.31**	
YOR153W	*PDR5*	**0.75**	**0.41**	1
YOR306C	*MCH5*		**0.34**	
YOR316C	*COT1*	**0.80**	**0.44**	1
YOR345C		**0.67**	**0.40**	
YOR359W	*VTS1*	**0.45**	**0.71**	
YOR382W	*FIT2*	**0.42**	**0.13**	1,2
YOR383C	*FIT3*	**0.31**	**0.10**	1,2
YPL019C	*VTC3*	**0.51**	**0.21**	

REPRESSED GENES				
ORF	GENE	-fold decrease		INFO
		at 8h	at 24h	
YPL030W	*TRM44*	**0.49**	**0.61**	
YPL068C		**0.34**		
YPL090C	*RPS6A*	**0.83**	**0.48**	4
YPL131W	*RPL5*	**0.73**	**0.47**	
YPL178W	*CBC2*	**0.88**	**0.26**	
YPL198W	*RPL7B*	**0.83**	**0.41**	4
YPR074C	*TKL1*	**0.71**	**0.42**	2,4
YPR136C			**0.47**	4
YPR145W	*ASN1*	**0.50**	**0.42**	4

1: Ion transport (FunCat 20.01.01)
2: Down-regulated by cryptolepine (Rojas et al, 2008)
3: Down-regulated tunicamycin (Kimata et al, 2006)
4: Repressed ESR gene (Gasch et al, 2000)
