# Supplementary material for: Depletion of yeast PDK1 orthologs triggers a stress-like transcriptional response
Source: BMC Genomics. 2015 Sep 21;16(1):719. doi: 10.1186/s12864-015-1903-8 (PMC4578605; doi:10.1186/s12864-015-1903-8)
Supplement: Additional file 3: Figure S1. — Semiquantitative analysis by RT-PCR of mRNA levels of a representative set of genes identified as altered by depletion of Pkh using microarrays analysis. RT-PCR was performed on total RNA isolated from the CML476 (WT) and SDP8 strains grown in YPD in the presence of doxycycline (100 μg/ml) for 8 and 24 h using specific sets of primers (Table 4). The numbers in brackets indicate the fold change (SDP8/WT) detected by microarrays for each gene at 8 and 24 h of incubation with doxycycline. RT-PCR of TUB2 is shown as a control. (PPTX 466 kb) [file 12864_2015_1903_MOESM3_ESM.pptx]

## Slide 1
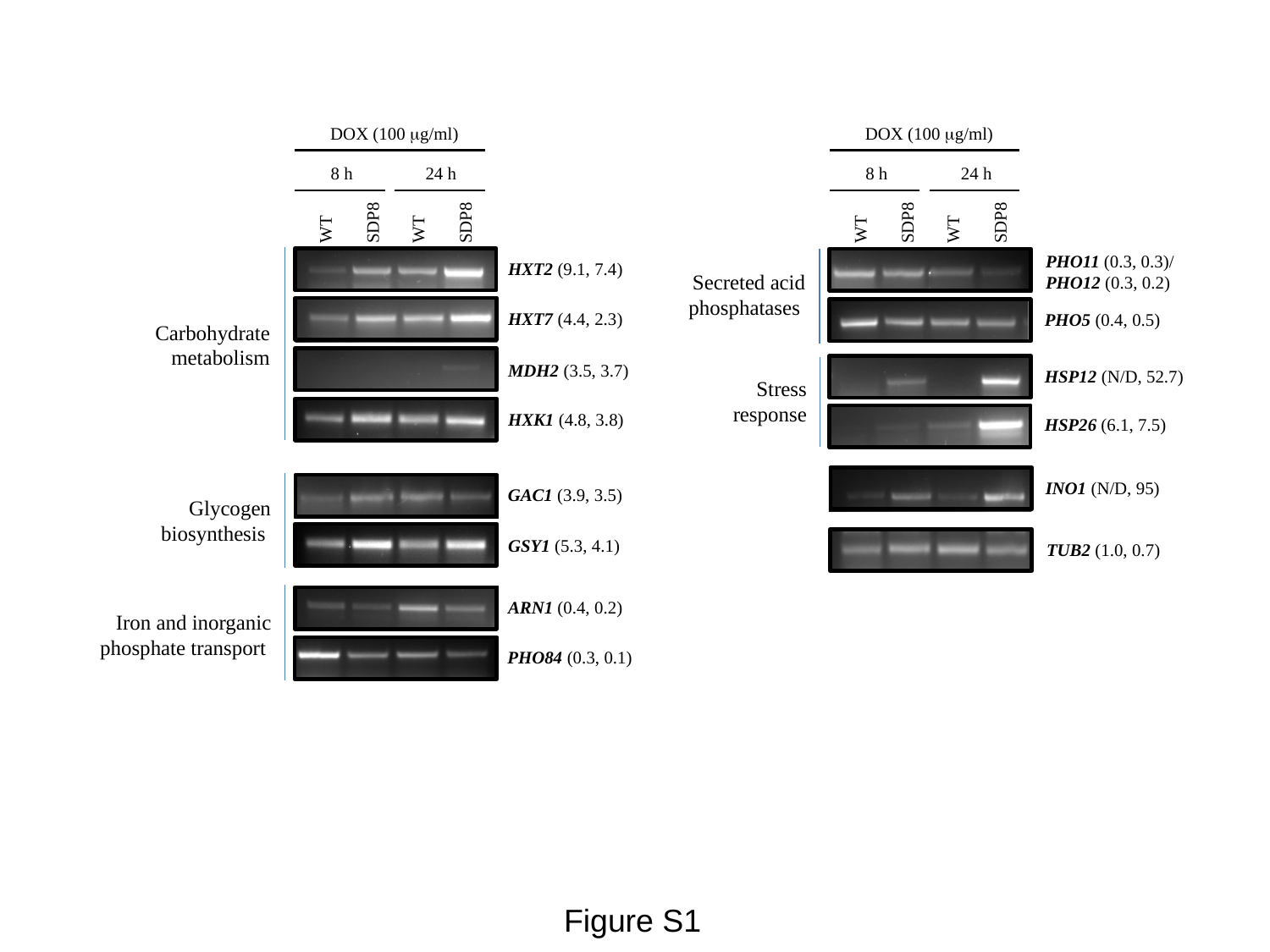

DOX (100 μg/ml)
8 h
24 h
SDP8
SDP8
WT
WT
DOX (100 μg/ml)
8 h
24 h
SDP8
SDP8
WT
WT
PHO11 (0.3, 0.3)/
PHO12 (0.3, 0.2)
Secreted acid
phosphatases
PHO5 (0.4, 0.5)
HXT2 (9.1, 7.4)
HXT7 (4.4, 2.3)
Carbohydrate
metabolism
MDH2 (3.5, 3.7)
HSP12 (N/D, 52.7)
Stress
response
HXK1 (4.8, 3.8)
HSP26 (6.1, 7.5)
INO1 (N/D, 95)
GAC1 (3.9, 3.5)
Glycogen
biosynthesis
GSY1 (5.3, 4.1)
TUB2 (1.0, 0.7)
ARN1 (0.4, 0.2)
Iron and inorganic
phosphate transport
PHO84 (0.3, 0.1)
Figure S1
