# Supplementary material for: Depletion of yeast PDK1 orthologs triggers a stress-like transcriptional response
Source: BMC Genomics. 2015 Sep 21;16(1):719. doi: 10.1186/s12864-015-1903-8 (PMC4578605; doi:10.1186/s12864-015-1903-8)
Supplement: Additional file 4: Table S3. — Expression changes of the ESR genes and their paralogs by depletion of Pkh. Data are the fold change of the expression values for ESR and non-ESR paralog genes in SDP8 cells incubated in the presence of doxycycline for 8 and 24 h compared to the values obtained in wild-type CML476 cells under the same treatment. (PDF 292 kb) [file 12864_2015_1903_MOESM4_ESM.pdf]

Supporting Table S3: Expression changes of the ESR genes
and their paralogs by depletion of Pkh.

ESR GENES			non-ESR Paralogs		
GENE	8 h (-fold change)	24 h (fFold change)	GENE	8 h (-fold change)	24 h (-fold change)
HXK1	4.84	3.86	*HXK2*	0.84	0.68
GLK1	1.84	2.02	*EMI2*	1.84	1.48
PGM2	4.20	3.66	*PGM1*	0.92	1.01
PFK26	N/A	1.51	*PFK27*	1.00	0.77
FBP26	N/A	N/A	*FBP1*	N/A	1.35
GPM2	1.16	0.82	*GPM1*<br>*GPM3*	1.03<br>0.68	0.72<br>0.61
GSY2	2.26	1.88	*GSY1*	5.35	4.14
GLG1	2.15	N/A	*GLG2*	N/A	N/A
NTH1	1.24	1.56	*NTH2*	N/A	N/A
GND2	N/A	4.00	*GND1*	0.69	0.51
GPD1	1.14	0.81	*GPD2*	0.86	0.96
CYC7	2.72	N/A	*CYC1*	N/A	N/A
TRX2	1.37	1.71	*TRX1*<br>*TRX3*	1.08<br>N/A	0.78<br>1.72
CTT1	1.17	1.12	*CTA1*	1.01	2.05
SOD1	1.13	1.34	*SOD2*	1.17	2.01
HYR1<br>*GPX1*	1.26<br>N/A	1.19<br>N/A	*GPX2*	N/A	1.26
GTT1	1.82	1.63	*GTT2*	N/A	N/A
PRX1<br>*TSA2*	1.85<br>N/A	2.86<br>2.24	*TSA1*	0.97	1.00
SSA3<br>*SSA4*	N/A<br>N/A	8.28<br>18.71	*SSA1*<br>*SSA2b*	0.68<br>0.58	1.04<br>0.92
SSE2	1.03	2.80	*SSE1*	0.74	0.86
HUL4	N/A	N/A	*UBA1*<br>*UBA2*<br>*RSP5*<br>*UBR1*	0.76<br>N/A<br>0.97<br>0.87	0.86<br>N/A<br>0.88<br>1.24
UBC5<br>*UBC8*	1.26<br>1.37	2.02<br>1.20	*UBC1*<br>*UBC4*<br>*UBC6*<br>*UBC9*<br>*UBC12*<br>*UBC13*	1.12<br>0.80<br>0.90<br>N/A<br>N/A<br>1.12	0.96<br>0.93<br>1.08<br>N/A<br>N/A<br>0.96
TOR1	N/A	0.76	*TOR2*	0.91	0.92
TPK1<br>*TPK2*	N/A<br>1.42	N/A<br>1.28	*TPK3*	0.78	0.75
PDE1	1.47	1.83	*PDE2*	0.88	0.56
