# Supplementary material for: Depletion of yeast PDK1 orthologs triggers a stress-like transcriptional response
Source: BMC Genomics. 2015 Sep 21;16(1):719. doi: 10.1186/s12864-015-1903-8 (PMC4578605; doi:10.1186/s12864-015-1903-8)
Supplement: Additional file 5: Figure S2. — Transcription factors mainly affected by depletion of Pkh. A: Transcription factors mainly involved in controlling the sets of genes found up- and down-regulated (left and right panel, respectively) when SDP8 were grown in the presence of doxycycline for 8 h. Transcription factors with more than 6 or 5 target genes (for up- and down-regulated, respectively) were selected. Only those with a ration of % of predicted/% of expected targets genes ≥ 3 are represented. B: As in A, but for the sets of genes found up- and down-regulated when cells were incubated with doxycycline for 24. Empty bars represent the % of up-regulated genes; filled bars denote the % of down-regulated genes and striped bars indicate the % of expected genes regulated by each transcription factor. Numbers on the top of empty bars denote the ratios % of up-regulated genes/% of expected number of genes regulated by each transcription factor. Numbers on the top of filled bars indicate the ratio of % of down-regulated genes/% of expected number of genes regulated by each transcription factor. (PPTX 91 kb) [file 12864_2015_1903_MOESM5_ESM.pptx]

## Slide 1
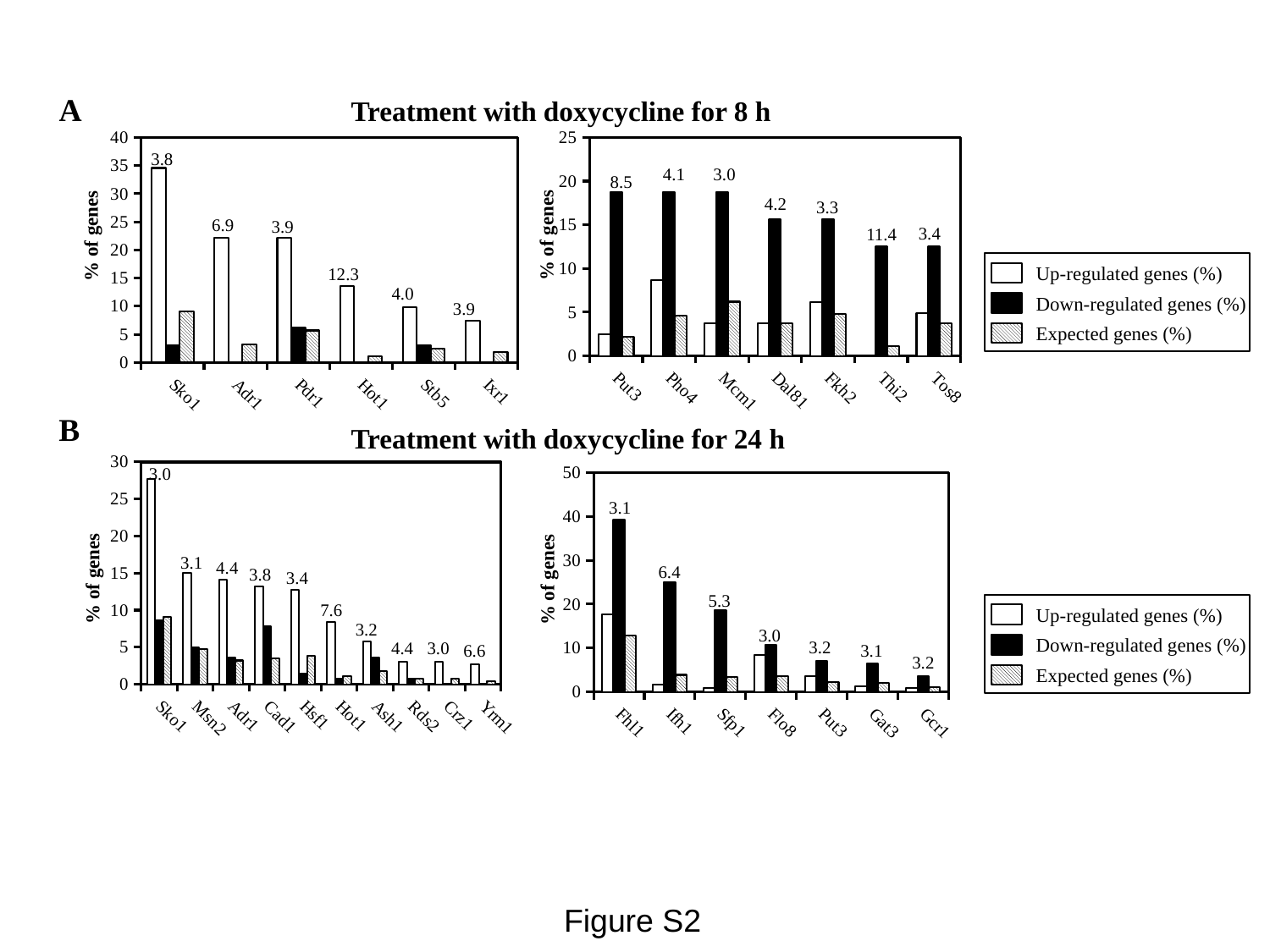

A
Treatment with doxycycline for 8 h
### Chart
| Category | % up-regulated genes | % down-regulated genes | Expected |
|---|---|---|---|
| Sko1 | 34.56790123456791 | 3.125 | 9.1 |
| Adr1 | 22.222222222222207 | 0.0 | 3.2 |
| Pdr1 | 22.222222222222207 | 6.25 | 5.7 |
| Hot1 | 13.580246913580252 | 0.0 | 1.1 |
| Stb5 | 9.876543209876546 | 3.125 | 2.5 |
| Ixr1 | 7.4074074074074066 | 0.0 | 1.9000000000000001 |3.8
6.9
3.9
12.3
4.0
3.9
### Chart
| Category | % up-regulated genes | % down-regulated genes | Expected |
|---|---|---|---|
| Put3 | 2.4691358024691366 | 18.75 | 2.2 |
| Pho4 | 8.64197530864197 | 18.75 | 4.6 |
| Mcm1 | 3.703703703703704 | 18.75 | 6.2 |
| Dal81 | 3.703703703703704 | 15.625 | 3.7 |
| Fkh2 | 6.1728395061728385 | 15.625 | 4.8 |
| Thi2 | 0.0 | 12.5 | 1.1 |
| Tos8 | 4.938271604938273 | 12.5 | 3.7 |4.1
3.0
8.5
4.2
3.3
3.4
11.4
% of genes
% of genes
Up-regulated genes (%)
Down-regulated genes (%)
Expected genes (%)
B
Treatment with doxycycline for 24 h
### Chart
| Category | % Up-regulated | % Down-regulated | % expected |
|---|---|---|---|
| Sko1 | 27.75330396475771 | 8.57 | 9.1 |
| Msn2 | 14.977973568281937 | 5.0 | 4.8 |
| Adr1 | 14.096916299559474 | 3.57 | 3.2 |
| Cad1 | 13.215859030837004 | 7.8599999999999985 | 3.5 |
| Hsf1 | 12.77533039647577 | 1.43 | 3.8 |
| Hot1 | 8.370044052863442 | 0.7100000000000002 | 1.1 |
| Ash1 | 5.7268722466960345 | 3.57 | 1.8 |
| Rds2 | 3.0837004405286352 | 0.7100000000000002 | 0.7000000000000002 |
| Crz1 | 3.0837004405286352 | 0.0 | 0.8 |
| Yrm1 | 2.6431718061674028 | 0.0 | 0.4 |3.0
3.1
4.4
3.8
3.4
7.6
3.2
4.4
3.0
6.6
### Chart
| Category | % Up-regulated | % Down-regulated | % expected |
|---|---|---|---|
| Fhl1 | 17.621145374449334 | 39.285714285714285 | 12.8 |
| Ifh1 | 1.762114537444934 | 25.0 | 3.9 |
| Sfp1 | 0.8810572687224667 | 18.571428571428573 | 3.5 |
| Flo8 | 8.370000000000003 | 10.71 | 3.6 |
| Put3 | 3.5242290748898677 | 7.1428571428571415 | 2.2 |
| Gat3 | 1.321585903083701 | 6.428571428571428 | 2.1 |
| Gcr1 | 0.9 | 3.5714285714285707 | 1.1 |3.1
6.4
5.3
3.0
3.2
3.1
3.2
% of genes
% of genes
Up-regulated genes (%)
Down-regulated genes (%)
Expected genes (%)
Figure S2
