# Supplementary material for: Depletion of yeast PDK1 orthologs triggers a stress-like transcriptional response
Source: BMC Genomics. 2015 Sep 21;16(1):719. doi: 10.1186/s12864-015-1903-8 (PMC4578605; doi:10.1186/s12864-015-1903-8)
Supplement: Additional file 6: Figure S3. — The transcriptional changes caused by heat shock are attenuated in Pkh-depleted cells. A: Graphical representation of the expression values (in log2) for the top 100 most up-regulated genes, after heat shock, in doxycycline-treated wild-type (WT) cells (○) and the corresponding value for the same genes in the SDP8 strain under the same condition (■). B: Similar representation for the top 100 most down-regulated genes after heat shock in doxycycline-treated wild-type (WT) cells (○) and the corresponding value for the same genes in the SDP8 strain under the same condition (■). C: Graphical representation of the expression values (in log2) for the top 100 most up-regulated genes by heat shock in doxycycline-treated SDP8 cells (■) and the corresponding value for the same genes in the wild-type (WT) strain under the same condition (○). D: Graphical representation of the expression values (in log2) for the top 100 most down-regulated genes, after heat shock, in doxycycline-treated SDP8 cells (■) and the corresponding value for the same genes in the wild-type (WT) strain under the same condition (○). (PPTX 87 kb) [file 12864_2015_1903_MOESM6_ESM.pptx]

## Slide 1
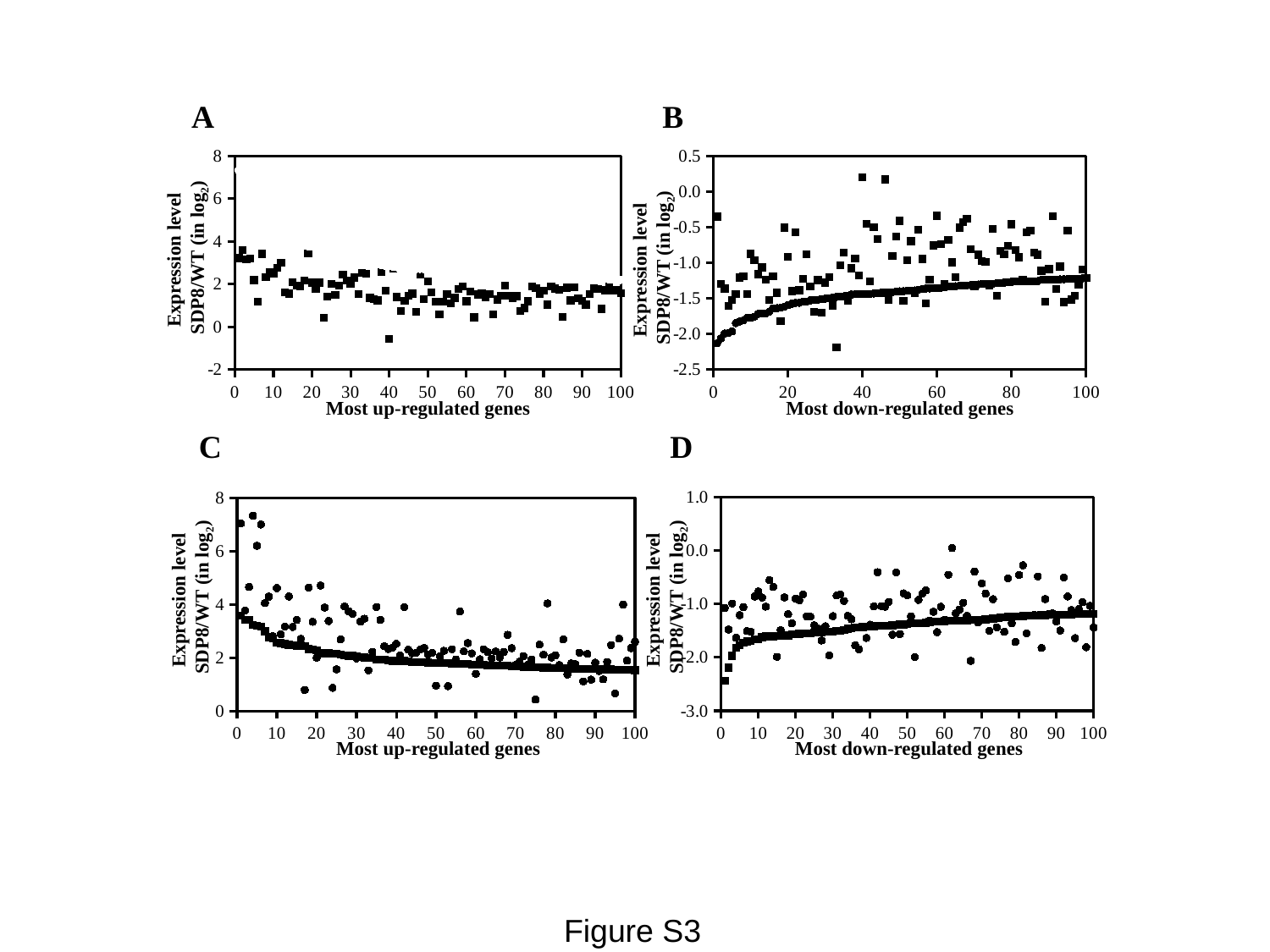

A
B
### Chart
| Category | WT | SDP8 |
|---|---|---|
### Chart
| Category | WT | SDP8 |
|---|---|---|Expression level
 SDP8/WT (in log2)
Expression level
 SDP8/WT (in log2)
Most up-regulated genes
Most down-regulated genes
C
D
### Chart
| Category | WT | SDP8 |
|---|---|---|
### Chart
| Category | WT | SDP8 |
|---|---|---|Expression level
 SDP8/WT (in log2)
Expression level
 SDP8/WT (in log2)
Most up-regulated genes
Most down-regulated genes
Figure S3
