# Supplementary material for: Depletion of yeast PDK1 orthologs triggers a stress-like transcriptional response
Source: BMC Genomics. 2015 Sep 21;16(1):719. doi: 10.1186/s12864-015-1903-8 (PMC4578605; doi:10.1186/s12864-015-1903-8)
Supplement: Additional file 7: Figure S4. — Analysis of the genes up-regulated by heat shock in a Pkh-dependent manner. A: Cluster analysis of the expression profiles of the sub-set of 52 genes that whose expression was up-regulated upon heat stress in a Pkh-dependent manner that were found already up-regulated in SDP8 cells treated with doxycycline for 24 h (“Depletion of Pkh”). Data was hierarchically clustered (complete linkage clustering, uncentered correlation) by means of the Gene Cluster (v. 2.11) software [57] and visualized with Java TreeView (v 3.0) [58]. B: Cluster analysis, performed as in A, but with the sub-set of 91 genes whose expression was also up-regulated upon heat stress in a Pkh-dependent manner but that were not found up-regulated in SDP8 cells treated with doxycycline for 24 h (“Depletion of Pkh”). (PPTX 79 kb) [file 12864_2015_1903_MOESM7_ESM.pptx]

## Slide 1
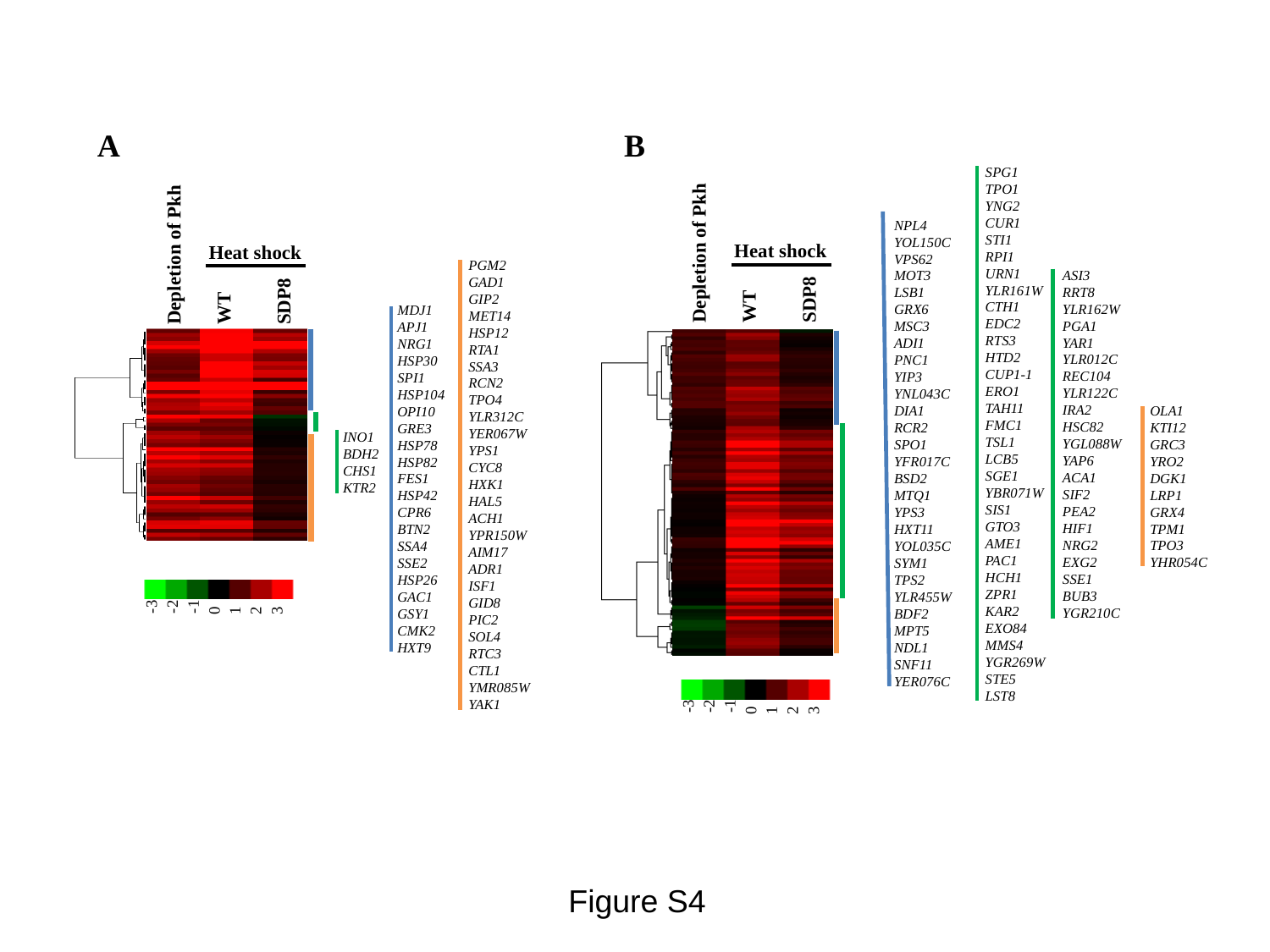

A
Heat shock
Depletion of Pkh
SDP8
WT
PGM2
GAD1
GIP2
MET14
HSP12
RTA1
SSA3
RCN2
TPO4
YLR312C
YER067W
YPS1
CYC8
HXK1
HAL5
ACH1
YPR150W
AIM17
ADR1
ISF1
GID8
PIC2
SOL4
RTC3
CTL1
YMR085W
YAK1
MDJ1
APJ1
NRG1
HSP30
SPI1
HSP104
OPI10
GRE3
HSP78
HSP82
FES1
HSP42
CPR6
BTN2
SSA4
SSE2
HSP26
GAC1
GSY1
CMK2
HXT9
INO1
BDH2
CHS1
KTR2
-3
-2
-1
0
1
2
3
B
SPG1
TPO1
YNG2
CUR1
STI1
RPI1
URN1
YLR161W
CTH1
EDC2
RTS3
HTD2
CUP1-1
ERO1
TAH11
FMC1
TSL1
LCB5
SGE1
YBR071W
SIS1
GTO3
AME1
PAC1
HCH1
ZPR1
KAR2
EXO84
MMS4
YGR269W
STE5
LST8
Heat shock
Depletion of Pkh
SDP8
WT
NPL4
YOL150C
VPS62
MOT3
LSB1
GRX6
MSC3
ADI1
PNC1
YIP3
YNL043C
DIA1
RCR2
SPO1
YFR017C
BSD2
MTQ1
YPS3
HXT11
YOL035C
SYM1
TPS2
YLR455W
BDF2
MPT5
NDL1
SNF11
YER076C
ASI3
RRT8
YLR162W
PGA1
YAR1
YLR012C
REC104
YLR122C
IRA2
HSC82
YGL088W
YAP6
ACA1
SIF2
PEA2
HIF1
NRG2
EXG2
SSE1
BUB3
YGR210C
OLA1
KTI12
GRC3
YRO2
DGK1
LRP1
GRX4
TPM1
TPO3
YHR054C
-3
-2
-1
0
1
2
3
Figure S4
