# Supplementary material for: Depletion of yeast PDK1 orthologs triggers a stress-like transcriptional response
Source: BMC Genomics. 2015 Sep 21;16(1):719. doi: 10.1186/s12864-015-1903-8 (PMC4578605; doi:10.1186/s12864-015-1903-8)
Supplement: Additional file 8: Table S4. — Genes whose up-regulation by heat stress was found to be dependent on the presence of Pkh. The values correspond to the -fold change of the expression levels triggered by heat shock in wild-type (WT) and SDP8 cells grown in the presence of doxycycline (100 μg/ml) for 24 h. Dependences on Pkh are defined as TD: totally dependent, SD: Strongly dependent, WD: weakly dependent. (PDF 74 kb) [file 12864_2015_1903_MOESM8_ESM.pdf]

Table S4. List of the genes whose up-regulation by heat stress was found to be dependent on the presence of Pkh. The values correspond to the -fold change in the expression levels in WT and SDP8 cells after the heat stress. Dependences on Pkh are defined as TD: totally dependent, SD: Strongly dependent, WD: weakly dependent.

Gene	WT	SDP8	Dependence
BTN2	160.54	9.30	TD
HSP30	131.59	11.94	TD
SSA4	127.73	8.98	TD
SPI1	73.67	9.24	TD
APJ1	26.12	4.54	TD
YJL144w	25.35	2.28	TD
ZPR1	25.23	10.69	SD
HSP78	24.77	4.99	TD
FES1	24.45	5.87	TD
HSP42	19.64	5.61	SD
MGA1	19.57	6.81	SD
LST8	16.52	7.94	SD
HSP26	16.41	3.07	TD
SIP4	15.92	2.93	TD
HSP104	15.15	4.27	SD
HSP82	14.98	3.80	SD
NRG1	14.89	3.67	TD
YAP6	14.72	4.49	SD
SWM1	13.42	4.19	SD
ASI3	13.31	3.42	SD
CUR1	12.50	4.18	SD
HSP12	11.17	1.33	TD
YNR068c	11.03	2.66	TD
YAR1	11.03	4.03	SD
TRP1	10.94	2.81	SD
URN1	10.68	3.79	SD
STE5	10.64	5.48	WD
SIS1	10.36	4.49	SD
STI1	10.17	4.06	SD
KAR2	10.16	4.92	SD
MUM3	9.57	2.89	SD
YGR210c	8.95	5.69	WD
YPR015c	8.88	5.54	WD
TSL1	8.35	2.56	SD
YHL008c	8.25	2.43	SD
MDJ1	7.53	2.35	SD
SIF2	7.25	3.23	SD
INO1	7.20	0.67	TD
ERO1	6.90	2.63	SD
GSY1	6.84	1.67	TD
SOL4	6.79	2.31	SD
RTS3	6.56	2.74	SD
EDC2	6.55	2.93	SD
SSE2	6.53	1.63	SD
ROG3	6.48	2.43	SD
YGR069w	6.43	3.04	SD
RTC3	6.32	2.26	SD
SSA3	6.28	1.49	TD
YLR162w	6.05	2.27	SD
REC104	6.05	2.90	SD
MET28	5.91	2.14	SD
CUP1-1	5.87	2.56	SD
YOL085c	5.85	3.39	WD
MMS4	5.72	3.69	WD

Gene	WT	SDP8	Dependence
CMK2	5.68	2.28	SD
HCH1	5.62	3.13	WD
TPO4	5.58	1.36	TD
EXG2	5.58	2.85	WD
RIM8	5.55	2.95	WD
YGR250c	5.54	2.59	SD
PEA2	5.51	2.89	WD
ADR1	5.49	1.51	SD
IRA2	5.49	2.41	SD
YHL041w	5.48	2.73	SD
OPI10	5.32	2.76	WD
YCRX11W	5.31	2.55	SD
GRE3	5.23	2.69	WD
YPR150w	5.18	1.68	SD
CPR6	5.17	1.84	SD
HSC82	5.16	2.30	SD
PAC1	5.12	2.92	WD
YGR269w	5.11	3.21	WD
TAH11	5.04	2.06	SD
EXO84	4.98	3.31	WD
SUE1	4.96	1.38	SD
YLR122c	4.93	2.36	SD
YLR012c	4.92	2.54	WD
YGL088w	4.89	2.33	SD
MTQ1	4.84	2.05	SD
GTO3	4.81	2.88	WD
GAC1	4.71	1.79	SD
YNL226w	4.56	3.00	WD
SGE1	4.51	2.60	WD
HIF1	4.44	1.68	SD
YLR161w	4.40	2.26	WD
SPG1	4.37	2.61	WD
YGR164w	4.34	2.72	WD
YNG2	4.24	2.65	WD
SER3	4.23	2.35	WD
YMR085w	4.07	2.22	WD
AST2	4.07	1.44	SD
YOL035c	4.05	2.26	WD
YAL064w	3.97	2.63	WD
UPS3	3.93	2.42	WD
CTH1	3.91	2.58	WD
YER076c	3.88	1.41	SD
RPI1	3.88	2.13	WD
FMP48	3.86	2.38	WD
RTA1	3.83	1.27	TD
HXT9	3.79	1.98	WD
YDR124w	3.72	2.46	WD
YPS3	3.72	1.89	WD
AME1	3.69	2.42	WD
YOL150c	3.68	1.28	TD
ISF1	3.63	1.45	SD
GAD1	3.62	1.05	TD
YGL074c	3.55	2.31	WD
HTD2	3.55	1.97	WD

Gene	WT	SDP8	Dependence
ADI1	3.54	1.50	SD
GRX4	3.49	1.76	WD
BDF2	3.42	1.20	TD
HXT11	3.41	2.11	WD
TPO1	3.40	2.22	WD
PNC1	3.39	1.40	SD
YBR071w	3.36	2.16	WD
RGS2	3.35	1.84	WD
YLR312c	3.32	1.37	SD
YDJ1	3.30	2.19	WD
PIC2	3.28	1.17	TD
LRP1	3.26	1.75	WD
PGA1	3.23	1.56	SD
BUB3	3.23	2.05	WD
YFR054c	3.18	2.07	WD
RCN2	3.15	1.29	TD
XBP1	3.14	1.50	SD
ECL1	3.14	1.82	WD
RCR2	3.13	1.33	SD
YER067w	3.12	1.40	SD
OM45	3.12	1.79	WD
VPS62	2.98	1.17	TD
TPM1	2.96	1.41	SD
FMC1	2.96	1.58	WD
PET18	2.88	1.58	WD
TPS2	2.84	1.85	WD
CPD1	2.84	1.68	WD
GIP2	2.80	1.08	TD
YLR455w	2.77	1.13	TD
SSE1	2.74	1.75	WD
CHS1	2.71	0.88	TD
DGK1	2.69	1.60	WD
ACA1	2.69	1.60	WD
GRC3	2.68	1.73	WD
MPT5	2.61	1.13	TD
DIA1	2.60	1.49	WD
PGM2	2.59	1.10	TD
YGR127w	2.56	1.40	WD
YTH1	2.56	1.34	WD
AAR2	2.51	1.34	WD
HXK1	2.51	1.40	WD
KTI12	2.50	1.41	WD
BSD2	2.49	1.48	WD

Gene	WT	SDP8	Dependence
SYM1	2.49	1.64	WD
MNN4	2.46	1.30	WD
LIN1	2.45	0.96	SD
AIM17	2.45	1.29	WD
HSP32	2.45	1.32	WD
YFR017c	2.43	1.31	WD
YRO2	2.42	1.48	WD
BDH2	2.41	0.86	SD
MSC3	2.40	1.37	WD
YJR129c	2.39	1.49	WD
HXT10	2.39	1.52	WD
SLI1	2.38	1.27	WD
RRT8	2.36	1.47	WD
YPS1	2.36	1.33	WD
MET14	2.36	1.09	SD
NRG2	2.31	1.38	WD
YAK1	2.30	1.47	WD
YBL029w	2.29	1.21	WD
SPO11	2.25	1.41	WD
NDL1	2.24	1.23	WD
HAL5	2.24	1.39	WD
YKE4	2.23	1.47	WD
LSB1	2.23	1.06	SD
LCB5	2.21	1.39	WD
MOT3	2.18	1.10	WD
SPO1	2.17	1.24	WD
YNL043c	2.17	1.33	WD
SNF11	2.17	1.25	WD
TPO3	2.16	1.10	WD
TEC1	2.15	0.94	SD
CYC8	2.13	1.23	WD
KTR2	2.11	0.93	SD
GRX6	2.11	1.22	WD
ARG81	2.11	1.34	WD
GID8	2.09	1.29	WD
YIP3	2.09	1.38	WD
NPL4	2.04	0.83	SD
CTL1	2.04	1.34	WD
YHR054c	2.04	1.08	WD
OLA1	2.03	1.28	WD
RAD16	2.02	1.07	WD
ACH1	2.02	1.34	WD
