# Supplementary material for: Depletion of yeast PDK1 orthologs triggers a stress-like transcriptional response
Source: BMC Genomics. 2015 Sep 21;16(1):719. doi: 10.1186/s12864-015-1903-8 (PMC4578605; doi:10.1186/s12864-015-1903-8)
Supplement: Additional file 9: Table S5. — Genes whose down-regulation by heat stress was found to be dependent on the presence of Pkh. The values correspond to the -fold change of the expression levels triggered by heat shock in wild-type (WT) and SDP8 cells grown in the presence of doxycycline (100 μg/ml) for 24 h. Dependences on Pkh are defined as TD: totally dependent, SD: Strongly dependent, WD: weakly dependent. (PDF 32 kb) [file 12864_2015_1903_MOESM9_ESM.pdf]

Table S5. List of the genes whose down-regulation by heat stress was found to be dependent on the presence of Pkh. The values correspond to the -fold change in the expression levels in WT and SDP8 cells after the heat stress. Dependences on Pkh are defined as TD: totally dependent, SD: Strongly dependent, WD: weakly dependent.

Gene	WT	SDP8	Dependence
TSA1	0.23	0.78	SD
RPL27B	0.24	0.41	WD
RPL25	0.25	0.39	WD
YGL102C	0.28	0.43	WD
RPL1B	0.28	0.44	WD
PAN5	0.29	0.54	WD
RPS13	0.30	0.51	WD
CHA1	0.30	0.48	WD
ERG2	0.33	0.70	SD
YHM2	0.33	0.53	WD
CIT2	0.34	0.67	SD
RPL40A	0.34	0.54	WD
PGA3	0.36	0.55	WD
AHP1	0.37	1.15	TD
GCV3	0.37	0.73	WD
PRY1	0.37	0.71	WD
RPL28	0.37	0.63	WD
YGR079W	0.37	1.12	TD
LYS21	0.38	0.64	WD
TRX1	0.38	0.75	SD
RPL29	0.38	0.62	WD
RPL16B	0.38	0.69	WD
RPL18A	0.39	0.59	WD
GPG1	0.39	0.79	SD
MRPL23	0.39	0.60	WD
SPE4	0.40	0.62	WD
ANT1	0.40	0.70	WD
YDR391C	0.40	0.74	WD
PDC1	0.40	0.77	WD
TPI1	0.41	0.70	WD
LYS20	0.42	0.73	WD
ALD6	0.42	0.67	WD
MRP17	0.42	0.68	WD
MMF1	0.42	0.79	WD
DIC1	0.43	0.68	WD
RPE1	0.43	0.67	WD
SKS1	0.43	0.65	WD
ADH2	0.44	0.66	WD
COF1	0.44	0.74	WD
ZRT1	0.44	0.67	WD
TOM40	0.44	0.70	WD
AOS1	0.44	0.74	WD
MTF1	0.45	0.66	WD
TRR1	0.45	0.71	WD
ADH1	0.45	0.70	WD
ASP3-3	0.45	1.08	SD
VTC1	0.45	0.71	WD
UBC5	0.45	0.88	WD
PDC5	0.45	0.73	WD
RSM24	0.45	0.75	WD
OAC1	0.45	0.84	WD
ENO1	0.45	0.74	WD
AIM45	0.45	0.71	WD
MOG1	0.46	0.79	WD
FIT2	0.46	0.78	WD
MET17	0.46	0.85	WD
YNL208W	0.46	0.81	WD
ASP3-4	0.47	0.98	SD
GCV1	0.47	0.81	WD
MRPL7	0.47	0.80	WD
SPC24	0.47	0.77	WD
MRPL38	0.47	0.75	WD
GRE2	0.47	0.83	WD
PGK1	0.48	0.94	WD
DSK2	0.48	0.79	WD
ATG33	0.48	1.04	SD
VBA1	0.48	0.79	WD
PFA4	0.48	0.80	WD
ASP3-1	0.49	1.11	SD
ASP3-2	0.49	1.02	SD
SCP1	0.49	0.90	WD
CDC36	0.49	0.91	WD
YDR154C	0.49	0.88	WD
CPR1	0.49	0.90	WD
TIM11	0.50	0.77	WD
MRPL40	0.50	0.77	WD
YGL039W	0.50	0.75	WD
PYC1	0.50	1.06	SD
