# Supplementary material for: Depletion of yeast PDK1 orthologs triggers a stress-like transcriptional response
Source: BMC Genomics. 2015 Sep 21;16(1):719. doi: 10.1186/s12864-015-1903-8 (PMC4578605; doi:10.1186/s12864-015-1903-8)
Supplement: Additional file 10: Table S6. — Major functional categories of genes up- and down-regulated by heat stress. The set of genes in each category is classified as affected (dependent) or unaffected (independent) by the absence of Pkh. (PDF 21 kb) [file 12864_2015_1903_MOESM10_ESM.pdf]

Table S6. Major functional categories of genes up- and down-regulated by heat stress. The set of genes in each category is classified as affected (dependent) or unaffected (independent) by the absence of Pkh.

Up-regulated by heat stress

	Total (433 genes)		Pkh-independent (240 genes)		Pkh-dependent (193 genes)	
FUNCTIONAL CATEGORY	Number of genes	p-value	Number of genes	p-value	Number of genes	p-value
**02 ENERGY**	36	1.86E-01	13	8.45E-01	23	1.82E-02
02.19 metabolism of energy reserves	14	1.99E-04	4	2.40E-01	10	6.40E-05
**14 PROTEIN FATE**	94	4.02E-01	42	8.80E-01	52	7.04E-02
14.01 protein folding and stabilization	28	4.92E-09	7	1.42E-01	21	1.30E-10
**32 CELL RESCUE, DEFENSE AND VIRULENCE**	89	2.04E-05	31	5.50E-01	58	8.35E-09
32.01 stress response	75	4.85E-05	27	4.22E-01	48	2.23E-07
32.01.07 unfolded protein response	23	2.04E-07	5	3.02E-01	18	2.29E-09

Down-regulated by heat stress

	Total (225 genes)		Pkh-independent (147 genes)		Pkh-dependent (78 genes)	
FUNCTIONAL CATEGORY	Number of genes	p-value	Number of genes	p-value	Number of genes	p-value
**01 METABOLISM**	73	5.84E-01	37	9.85E-01	36	8.68E-03
01.01 amino acid metabolism	27	4.13E-05	11	1.51E-01	16	1.90E-06
01.01.06 metabolism of the aspartate family	13	1.26E-05	4	1.52E-01	9	1.11E-06
01.01.06.02 metabolism of asparagine	5	1.47E-05	1	2.19E-01	4	4.99E-06
01.01.06.02.02 degradation of asparagine	4	9.05E-06	0	ND	4	1.25E-07
01.05 C-compound and carbohydrate metabolism	36	4.44E-02	17	6.17E-01	19	1.73E-03
**02 ENERGY**	34	1.94E-04	14	2.88E-01	20	1.75E-06
02.16 fermentation	9	9.80E-04	3	1.55E-01	5	7.03E-04
02.16.01 alcohol fermentation	6	4.47E-05	1	3.74E-01	5	3.10E-06
**12 PROTEIN SYNTHESIS**	97	6.04E-43	82	3.17E-47	15	5.22E-03
12.01 ribosome biogenesis	95	9.03E-61	80	7.30E-62	15	3.57E-05
12.01.01 ribosomal proteins	90	3.49E-69	75	7.26E-67	15	6.16E-07
12.04.02 translation elongation	6	9.95E-04	6	1.01E-04	0	ND
